# Development of a repetitive traumatic brain injury risk function based on real-world accident reconstruction and wavelet packet energy analysis

**DOI:** 10.3389/fbioe.2025.1548265

**Published:** 2025-03-26

**Authors:** Tao Xiong, Qinghang Luo, Qiuju Chen, Liangliang Shi, Aowen Duan, Shengxiong Liu, Kui Li

**Affiliations:** ^1^ College of Medical Information, Chongqing Medical University, Chongqing, China; ^2^ School of Pharmacy and Bioengineering, Chongqing University of Technology, Chongqing, China; ^3^ China Automotive Engineering Research Institute Co., Ltd., Chongqing, China; ^4^ Department of Medical Engineering, Daping Hospital, Army Medical University, Chongqing, China

**Keywords:** accident reconstruction, wavelet packet transform, survival analysis, injury risk function, repetitive traumatic brain injury tolerance

## Abstract

Existing evaluation criteria for pedestrian head impact injuries focus only on single impacts, with less attention given to repetitive traumatic brain injury (rTBI), which is common in motor vehicle collisions, falls, and sports. Improving pedestrian collision protection safety requires a complete understanding of the tolerance of the repeated collisions of the human brain to injury. Therefore, this study aimed to collect data from 72 pedestrian collisions that were reconstructed using MADYMO and THUMS finite element head models (version 4.0.2). The evaluation metrics for rTBI were developed by integrating brain injury criteria based on time-domain features, including the head injury criterion (HIC), brain injury criterion (BrIC), diffuse axonal multi-axial general evaluation (DAMAGE), and maximum principal strain (MPS), with frequency-domain features obtained from wavelet packet transform energy analysis of head motion responses. The proposed brain tolerance for mild and severe rTBI was estimated through parametric survival analysis and presented as injury risk curves based on the selected injury metrics. The results showed a significant difference in brain injury tolerance between repetitive and single collisions. For the 50% probability of mild and severe brain injury in real accidents, the thresholds for rTBI metrics based on BrIC and DAMAGE were 1.085 and 1.513 and 0.494 and 0.678, respectively, all higher than the thresholds of single-impact reported in previous studies. However, the thresholds for repetitive head injury criteria based on MPS were 0.604 and 0.838, which were lower than the thresholds of single impact reported in previous studies, implying that the prediction of tolerance to repetitive brain more consistent with tissue-level than head kinematics level. This study developed injury risk functions (IRFs) for rTBI by integrating the amplitude–frequency characteristics of head responses and brain injury criteria. This knowledge further provides crucial support for understanding the tolerance to rTBI and enhancing pedestrian safety.

## 1 Introduction

Traumatic brain injury (TBI) has always been a significant public health issue. Among various types of traffic accidents, pedestrian collisions are considered one of the most typical and high-risk. According to statistics from the World Health Organization (WHO, 2023), approximately 1.19 million people die in road traffic accidents each year, with 21% of these deaths resulting from pedestrian–vehicle collisions. In pedestrian collision incidents, most pedestrians struck by vehicles subsequently come into contact with the ground, so it is essential to consider the risk of injury caused by ground impact (Guillaume et al., 2015; Shang et al., 2020). When designing safer vehicle front-end structures to protect these vulnerable road users, it is crucial to have a deep understanding of head biomechanical tolerance in real-world pedestrian collisions.

Over the past half-century, numerous studies have focused on developing injury metrics associated with external mechanical loads and establishing injury risk functions (IRFs) to assess human biomechanical tolerance to TBI (Gurdjian et al., 1966; Takhounts et al., 2013; Wu et al., 2020). These injury metrics are primarily based on empirical formulas or fundamental principles of mechanics related to external head kinematics, which are further derived into IRFs. Historically, these IRFs mainly targeted time-domain information for a single impact. The earliest research on the injury mechanisms of concussion and skull linear fractures was conducted through cadaveric experiments and led to the development of the Wayne State Tolerance Curve (WSTC; Gurdjian et al., 1966). This curve primarily considers linear acceleration and duration of the head kinematics to describe the head injury. Subsequently, a logistic regression model was used to establish an IRF for the head injury criterion (HIC, Prasad and Mertz, 1985) based on the resultant linear acceleration of the head. In recent years, the development methods of IRFs have gradually shifted toward using survival analysis (Petitjean et al., 2009; Petitjean and Trosseille, 2011; Wu et al., 2021; Wu et al., 2022), and brain injury criteria have also been established based on rotational kinematics, such as brain injury criterion (BrIC, Takhounts et al., 2013), universal brain injury criterion (UBrIC, Gabler et al., 2018), and diffuse axonal multi-axial general evaluation (DAMAGE, Gabler et al., 2019), for assessing injury metrics. With a deeper understanding of brain injury mechanisms, it is recognized that given the incompressibility of brain tissue, deformation due to rotating head kinematics is the main cause of brain injury (Alshareef et al., 2020; Gennarelli et al., 1972). The censoring status (e.g., exact/uncensored, left, and right) provided by survival analysis improves the reliability and accuracy of data, while a well-fitted IRF distribution ensures zero risks of injury for zero stimuli (Di Domenico and Nusholtz, 2005; Jean et al., 2014). However, these kinematic-based and tissue-level metrics only represent the tolerance to brain injury in single collisions. The metric and tolerance for repetitive traumatic brain injury (rTBI) caused by pedestrian collisions have yet to be reliably established through data and methods.

Several innovative approaches have been proposed to develop assessment metrics for rTBI through kinematic events. For instance, metrics such as risk-weighted exposure (RWE, Stemper et al., 2019) and cumulative head impact indices (CHIIs, Daneshvar et al., 2023) have been developed to evaluate repetitive mild traumatic brain injuries (rmTBIs) by quantifying the number of head impacts and the intensity of linear and rotational accelerations. However, these studies often employ linear superposition methods within a nonlinear injury framework and limitations in the fidelity of data acquisition (O'Connor et al., 2017). Current impact tests mainly rely on measuring head kinematic responses of dummies equipped with internal sensor arrays (Kanianthra et al., 1996). These dummies simulate the connective tissue between the skull and the brain using discrete mechanical components, such as springs and dampers, while brain deformation is represented as a resonant mechanical system operating at specific natural frequencies (Laksari et al., 2015; Wu et al., 2020). However, it is crucial to recognize that the time and frequency domains of head response can collectively describe the characteristics of brain injury (Laksari et al., 2018; Li et al., 2024). The maximum strain induced by brain surrogate materials is closely related to the frequency characteristics of the kinematics applied to the head (Laksari et al., 2015). Although time-domain correlated information is vital for understanding subsequent injuries, it is not the sole determining factor.

This study aims to develop a novel method to evaluate human tolerance to repetitive mild and severe TBI by combining brain injury criteria with wavelet packet transform (WPT) characteristics. First, we used MADYMO and finite element (FE) modeling techniques to reconstruct the pedestrian collision injury data. Based on six degrees of freedom (6DOF) kinematics data of pedestrian collision, the frequency domain characteristics of head motion response are obtained by WPT. This method has strong time–frequency localized decomposition ability and can accurately extract the features of high- and low-frequency components. Then, the rTBI metrics were established by combining the brain injury criteria and tissue-level index, and their correlation was evaluated. Second, we evaluated the discriminatory power of the rTBI metrics included in the survival analysis using the area under the receiver operating characteristic (ROC). Finally, we estimated human tolerance to rTBI by developing IRFs through survival analysis using the selected kinematic and tissue-level metrics. The findings of this study will provide critical scientific evidence for reinforcing rTBI criteria and formulating pedestrian safety guidelines.

## 2 Materials and methods

### 2.1 Repetitive traumatic brain injury data

The data in this study are based on the depth reconstruction of pedestrian collision accidents. A total of 72 accident cases were collected from the video database of the Institute of Transportation Medicine at the Chinese Army Medical University and the Changsha Vehicle Accident Depth Investigation Database. All of them were pedestrian collisions, and pedestrians collided with the ground after colliding with the vehicle during the collision. The selection criteria for the accident cases and their details are provided in Supplementary Tables A1, A2.

### 2.2 Accident reconstruction

**FIGURE 1:**
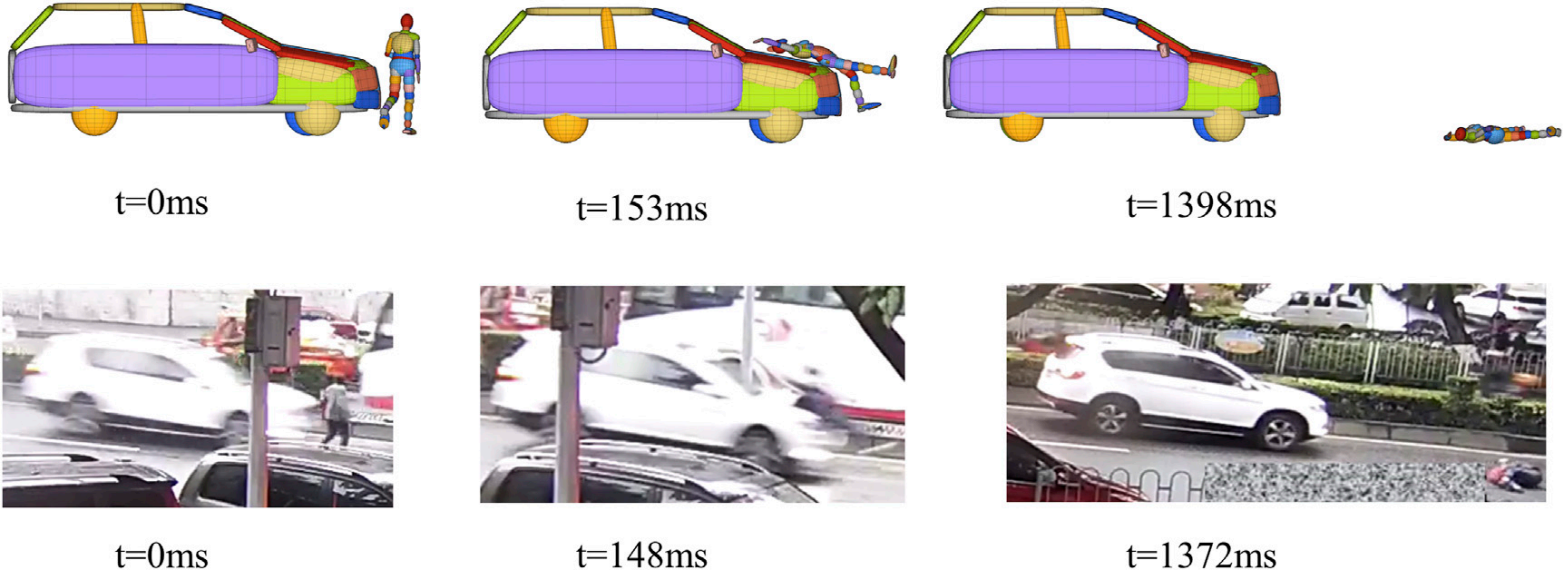
Comparison of reconstruction kinematics and video records.

Two methods were employed to calculate the pre-impact speed of vehicles in pedestrian collisions, namely, video frame rate analysis and an initial estimate based on the kinetic energy theorem (the impact speed *v* (km/h) is given by $v=3.6\times \sqrt{2\mu gL}$ , where *μ* is the friction coefficient, g = 9.8 m/s^2^, and *L* is the braking distance; Li et al., 2021). Using MADYMO software, multibody modeling was conducted to reconstruct the kinematics of vehicles and pedestrians in the 72 selected cases (Figure 1). Each multibody vehicle model’s geometry was developed based on blueprints or actual measurements of the respective accident vehicles, while the stiffness characteristics of the components were derived from crash block test data and subsystem test ratings reported in previous studies (Peng et al., 2014; Li et al., 2021). The pedestrian multibody models were scaled to match the height and weight of the victims.

**FIGURE 2:**
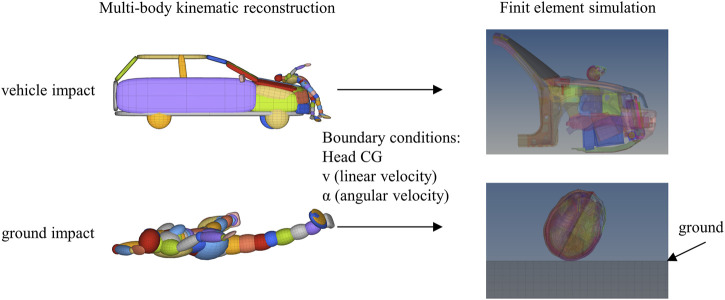
THUMS finite element head model (version 4.0.2). Vehicle and ground collision boundary condition loading mode.

The initial conditions for the head prior to impact with the vehicle and the ground were defined using the output data from multi-body kinematic reconstruction (Figure 2). These included the head’s triaxial linear and angular velocities and the relative center of gravity position to the impact points. Among the 72 cases, the vehicle types involved mainly consisted of SUVs, sedans, and MPVs. To match the FE vehicle models as closely as possible to the dimensions of the actual accident vehicles, three different FE vehicle front models were utilized for the head-to-vehicle impact simulations (Shi et al., 2018). The ground was simulated as a rigid asphalt surface. All FE simulations were conducted using LS-DYNA software (Ver. MPP R9.3.0, LSTC). In MADYMO, the body is used to determine the head kinematics; in order to obtain the calculation results quickly, only the head model extracted from the THUMS AM50 V4.0 human body model was used for the head impact simulations. The THUMS head model has been validated for its high biofidelity (Iwamoto et al., 2015; Shi et al., 2020), ensuring reliability and accuracy in this study.

### 2.3 Head injury metrics

To assess head injury, standard brain injury criteria were utilized, including HIC based on linear acceleration, BrIC based on angular velocity, and DAMAGE based on rotational acceleration. The constraints and critical values for these injury metrics are adapted from the corresponding studies (Prasad and Mertz, 1985; Takhounts et al., 2013; Gabler et al., 2019), and a brief description of each metric is provided in “Supplementary Appendix B.” The calculation of these three brain injury criteria was automated using the MATLAB code (MATLAB v8.4.0, the MathWorks Inc., Natick, MA). The brain tissue level-based injury criterion, MPS, was calculated using the LS-DYNA code.

### 2.4 Development of repetitive traumatic brain injury metrics

The head impact causes mechanical resonance in the brain, amplifying the relative motion between the brain and the skull, and brain deformation is most sensitive in the low-frequency range. Moreover, shear strain in brain tissue caused by low-frequency exercise is considered to be the main cause of concussion and diffuse axonal injury (Laksari et al., 2015; Laksari et al., 2018). Therefore, we adopt WPT, also known as an optimal sub-band tree structure, which is an efficient method to solve non-stationary signals and low-frequency transient effects (Wang et al., 2019). It further enhances the performance of the wavelet transform by subdividing the high- and low-frequency components, thus providing an improved time–frequency resolution to fully capture the frequency characteristics of head movements. A detailed description is provided in Supplementary Appendix B.

In pedestrian collision analysis, representing repetitive brain damage is crucial, so it is necessary to preserve the traces of brain injury caused by the pedestrian–vehicle impact to assess the severity caused by the second impact with the ground. There is a significant correlation between the wavelet packet transform energy (WPTE) characteristics in the frequency domain and the head injury criteria in the time domain (Li et al., 2024). We have utilized frequency-domain characteristics to establish a connection between pedestrian head impacts with vehicles and ground collisions. The contribution of ground impact and vehicle impact to brain injury is independent and can be accumulated. Based on the brain injury assessment value calculated from the first impact, the ratio of the frequency-domain total energy during the ground impact to the total energy of the entire collision process was used as a cumulative weighting factor. This factor was multiplied by the brain injury assessment value of the second impact and then added to the initial value, thereby integrating frequency-domain information into the traditional time-domain injury assessment framework. Based on the WPTE, we developed WPTE_HIC, WPTE_BrIC , WPTE_DAMAGE, WPTE_MPS rTBI metrics, which are formulated as follows:

$$\text{WPT}E-\text{HIC}={\text{HIC}}_{\text{car}}+\frac{\sum_{j=0}^{2^{i}-1}\left(\int {\left|{a_{\text{ground}}}_{i,j}\left(t\right)\right|}^{2}\text{dt}\right)}{\sum_{j=0}^{2^{i}-1}\left(\int {\left|a_{i,j}\left(t\right)\right|}^{2}\text{dt}\right)}{\text{HIC}}_{\text{ground}},$$ (1)

$$\text{WPT}E-\text{BrIC}={\text{BrIC}}_{\text{car}}+\frac{\sum_{j=0}^{2^{i}-1}\left(\int {\left|{\alpha_{\text{ground}}}_{i,j}\left(t\right)\right|}^{2}\text{dt}\right)}{\sum_{j=0}^{2^{i}-1}\left(\int {\left|\alpha_{i,j}\left(t\right)\right|}^{2}\text{dt}\right)}{\text{BrIC}}_{\text{ground}},$$ (2)

$$\text{WPT}E-\text{DAMAGE}={\text{DAMAGE}}_{\text{car}}+\frac{\sum_{j=0}^{2^{i}-1}\left(\int {\left|{\alpha_{\text{ground}}}_{i,j}\left(t\right)\right|}^{2}\text{dt}\right)}{\sum_{j=0}^{2^{i}-1}\left(\int {\left|\alpha_{i,j}\left(t\right)\right|}^{2}\text{dt}\right)}{\text{DAMAGE}}_{\text{ground}},$$ (3)

$$\text{WPT}E-\text{MPS}={\text{MPS}}_{\text{car}}+\frac{\sum_{j=0}^{2^{i}-1}\left(\int {\left|{\alpha_{\text{ground}}}_{i,j}\left(t\right)\right|}^{2}\text{dt}\right)}{\sum_{j=0}^{2^{i}-1}\left(\int {\left|\alpha_{i,j}\left(t\right)\right|}^{2}\text{dt}\right)}{\text{MPS}}_{\text{ground}},$$ (4)

where *i* is the number of decomposition levels and *j* is the number of sub-bands for the corresponding level. $a_{i,j}\left(t\right)$ is the resultant linear acceleration response of the head during the whole pedestrian collision, and $\alpha_{i,j}\left(t\right)$ is the resultant angular acceleration response of the head during the whole pedestrian collision. ${HIC}_{car}$ , ${BrIC}_{car}$ , ${DAMAGE}_{car}$ , and ${MPS}_{car}$ represent the assessment of brain injury in vehicular contact, while ${HIC}_{ground}$ , ${BrIC}_{ground}$ , ${DAMAGE}_{ground}$ , ${MPS}_{ground}$ , ${a_{ground}}_{i,j}\left(t\right)$ , and ${\alpha_{ground}}_{i,j}\left(t\right)$ represent the evaluation of brain injury in ground contact.

### 2.5 Statistical analysis

The prediction of the kinematics-based metrics on the tissue-level metrics (MPS) was assessed using the coefficient of determination (*R* ^2^) and the normalized root mean square error (NRMSE, Takhounts et al., 2013; Gabler et al., 2019), calculated as RMSE divided by the mean of the dependent variable. 1-NRMSE values are reported, which range from 0 to 1, with higher values indicating a better fit. ROC curves and the area under the curve (AUC) were then used to evaluate the classification ability of rTBI metrics for pedestrian collisions.

The risk curve of rTBI was obtained through parametric survival analysis following the procedure outlined by the International Organization for Standardization (ISO/TS 18506:2014). These curves are derived from the best metrics of three variables: BrIC, DAMAGE, and MPS. These variables are considered responses and include the level of kinematics and tissue. All response data are uncensored observational data. According to the International Organization for Standardization (ISO/TR 19222:2021), injury outcomes were divided into rmTBI with AIS scores of 2 and 3 and repetitive severe traumatic brain injury (rsTBI) with AIS scores of 4 and 5 (See Supplementary Table SA2). The censored data are divided according to the injury property description, and data points that occur precisely within the defined injury cases are considered uncensored. Those that occur after the measured stimulus are called left-censored, whereas those that occur before the measured stimulus are called right-censored. In the rmTBI cases, AIS scores below 2 are considered right-censored, scores above 3 are considered left-censored, and scores equal to 2 and 3 are considered uncensored. In the rsTBI cases, AIS scores below 4 are considered right-censored, scores above 5 are considered left-censored, and scores equal to 4 and 5 are considered uncensored.

The cumulative distribution functions (CDFs) for Weibull, log-normal, and log-logistic distributions are as follows:

$$\text{Loglogistic}:F\left(x;\alpha ,\beta \right)=\frac{1}{1+{\left(\frac{x}{\alpha }\right)}^{-\beta }},$$ (5)

$$\text{Lognormal}:F\left(x;\mu ,\beta \right)=\frac{1}{2}\left(1+\mathrm{erf}\left(\frac{\ln\left(x\right)-\mu }{\beta \sqrt{2}}\right)\right),$$ (6)

$$\text{Weibull}:F\left(x;\alpha ,\beta \right)=\left\{\begin{array}{cc} 1-e^{-{\left(\frac{x}{\alpha }\right)}^{\beta }} & x\geq 0\\ 0 & x<0 \end{array}\right.,$$ (7)

where *α* is the shape parameter, *β* is the scale parameter, μ is the location parameter, and *x* is the index of the rTBI response. For the lognormal distribution, “erf” is expressed as the standard Gaussian error function as follows:

$$\mathrm{erf}\left(t\right)=\frac{2}{\sqrt{\pi }}\int_{0}^{t}e^{-x^{2}}dx.$$ (8)

Based on the Akaike information criterion (AIC) and quality index (QI), Weibull, log-normal, and log-logistic distributions were evaluated, and the candidate metric probability distribution with the lowest AIC is determined as the optimal distribution function among the candidates. The positive and negative 95% confidence intervals (CIs) of the estimated values of the best response variables were calculated, and the normalized confidence interval sizes (NCISs) quantified the average CI ( u ) curve and upper CI ( $U_{CI}$ ) and lower CI ( $L_{CI}$ ) curve discrete probabilities of the points in terms of NCIS as follows:

$$\text{NCIS}={\left(\frac{U_{\text{CI}}-L_{\text{CI}}}{u}\right)}_{\text{Ris}k_{\text{Level}}\%}.$$ (9)

The lower NCIS correlates more closely with the confidence interval at the chosen probability level, which is calculated at the seven risk levels, namely, 5%, 10%, 25%, 50%, 75%, 90%, and 95%. The DFBETA statistics were used to identify cases with excessive effects. Distributional assumptions were checked using Q–Q plots to test distributions graphically (“Q” stands for quantile) and plot the cumulative risk calculated from survival analysis for a given distribution against the cumulative risk calculated from non-parametric maximum likelihood estimation (NPMLE) for consistency assessment. All survival statistical analyses described above were performed using RStudio software (R, v.4.3.3).

## 3 Results

### 3.1 Assessment of tissue-level and kinematics-based rTBI metrics

**FIGURE 3:**
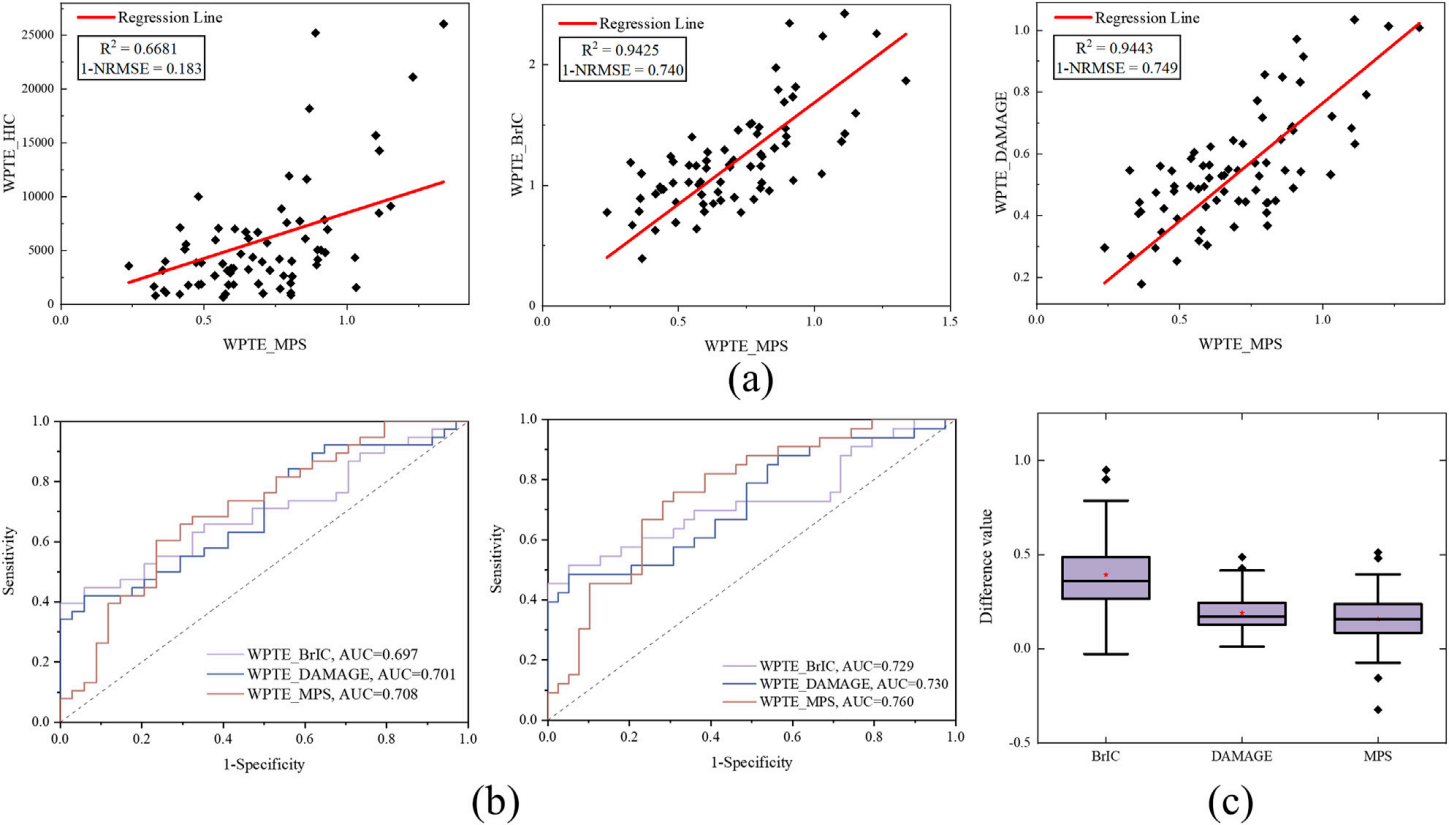
**(a)** Correlation and metric accuracy analysis of rTBI kinematics-based metrics WPTE_HIC, WPTE_BrIC, and WPTE_DAMAGE relative to the tissue-level rTBI metrics WPTE_MPS. **(b)** ROC curve of rTBI metrics; rmTBI is shown on the left, and rsTBI is shown on the right. **(c)** Differences between the assessment indicators in this study and existing criteria for real pedestrian collisions. For each box, the central band is the median, and the central ‘pentagram’ marker is the mean.

All pedestrian collision data (n = 72, Supplementary Table SA3) were used to establish rTBI evaluation indicators according to Equations 1-4, and the correlation analysis results based on kinematic indicators (BrIC, DAMAGE, and HIC) and tissue level indicators (MPS) are shown in Figure 3A. The correlation and goodness-of-fit between WPTE_DAMAGE and WPTE_MPS are high (*R* ^2^ = 0.9443 and 1-NRMSE = 0.749), followed by WPTE_BrIC (*R* ^2^ = 0.9425 and 1-NRMSE = 0.740). However, the correlation and goodness-of-fit for WPTE_HIC are poor (*R* ^2^ = 0.6681 and 1-NRMSE = 0.183); therefore, it is not recommended. Considering this, the rTBI metrics based on BrIC, DAMAGE, and MPS were mainly used in this study to develop IRFs. Figure 3B shows the ROC curves for the classification of real pedestrian collisions, with WPTE_MPS exhibiting the best accuracy, while WPTE_DAMAGE and WPTE_BrIC show similar accuracy. Finally, the differences between the rTBI metrics in this study and the existing brain injury criterion assessment value were compared (Figure 3C).

### 3.2 Statistical modeling: development of repetitive brain injury risk curves

#### 3.2.1 Assessment of the injury risk function distribution assumption

**FIGURE 4:**
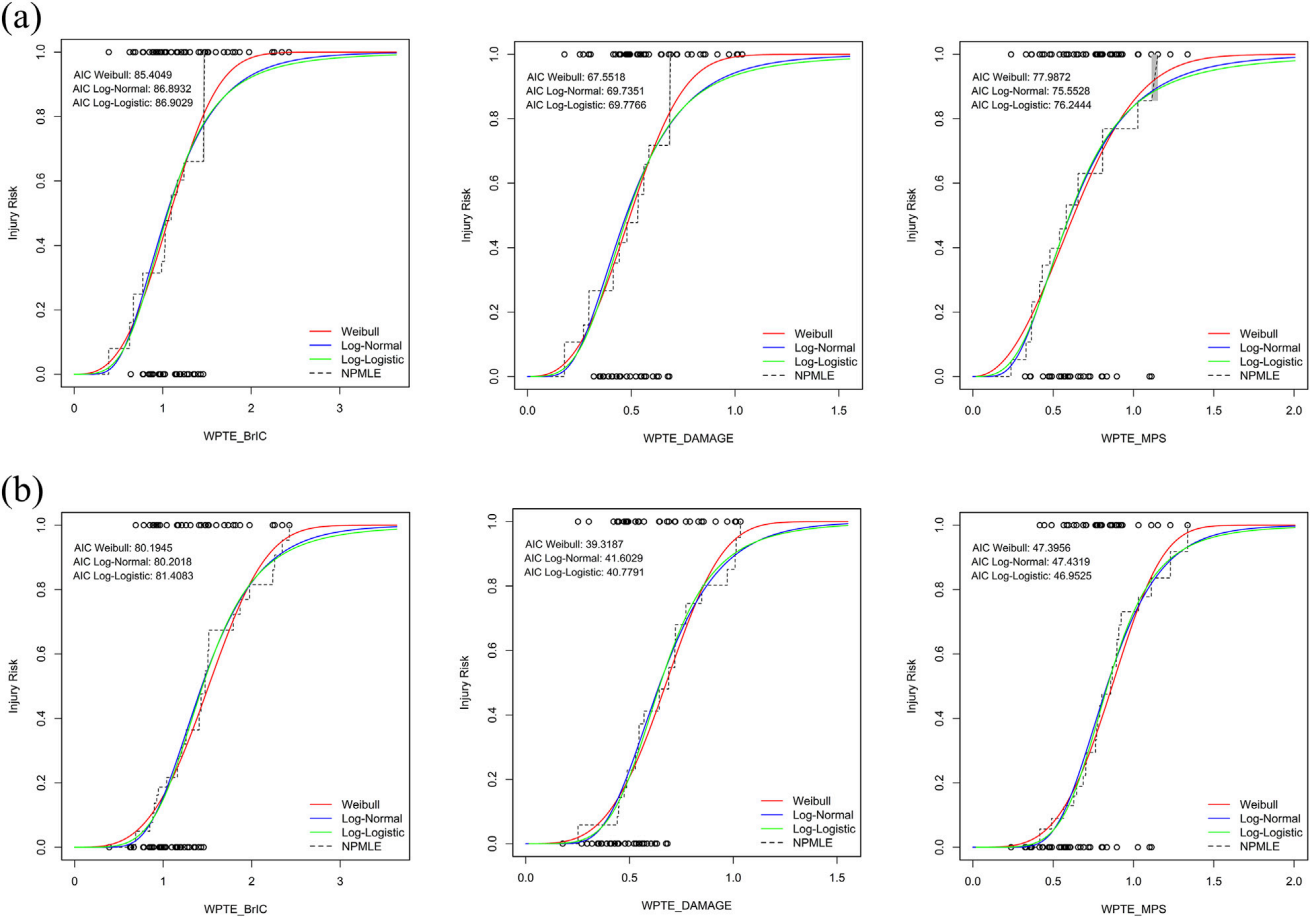
Comparison of the distribution of injury risk assumptions for rTBI metrics. Data points by censoring (circle): **(a)** rmTBI and **(b)** rsTBI.

Substantially different results can be obtained when extrapolating outside the range of the observed data. In order to recommend the most suitable method for predicting the distribution of IRFs, we developed three CDFs based on rmTBI and rsTBI metrics using survival analysis: Weibull distribution, log-logistic distribution, and log-normal distribution (Figure 4). Evaluation using the AIC and NPMLE methods showed that the Weibull distribution had the lowest AIC values for WPTE_BrIC and WPTE_DAMAGE, making it the optimal IRF choice for these two metrics. For rmTBI, log-normal distribution was found to be the optimal IRF for WPTE_MPS, while for rsTBI, log-logistic distribution was preferred. Overall, the IRF of WPTE_DAMAGE exhibited better quality among all metrics with the lowest AIC value.

#### 3.2.2 Assessment of influential observations and check on the distribution assumption

When comparing IRF distribution assumptions in rTBI metrics, Weibull distributions are the most optimal distributions selected based on AIC values. We examined the fitting results of the best distribution assumption through influential observations, Q–Q plots, and the NPMLE method (Supplementary Figure C1, C2). DFBETA statistics identified influential samples in each rTBI metric dataset. However, these samples did not significantly alter the IRF and were retained when constructing the IRFs. The Q–Q plot showed a slight deviation between percentile values of the optimal distribution and corresponding percentile values of the rTBI metrics for WPTE_DAMAGE and WPTE_MPS, with almost all points falling on a straight line; however, WPTE_BrIC performed poorly. In validating model fitting using the NPMLE method, WPTE_DAMAGE had the highest log-likelihood value and the most flexible fit, with its survival analysis cumulative risk graph closely resembling that of NPMLE.

### 3.3 Injury threshold and quality index

**FIGURE 5:**
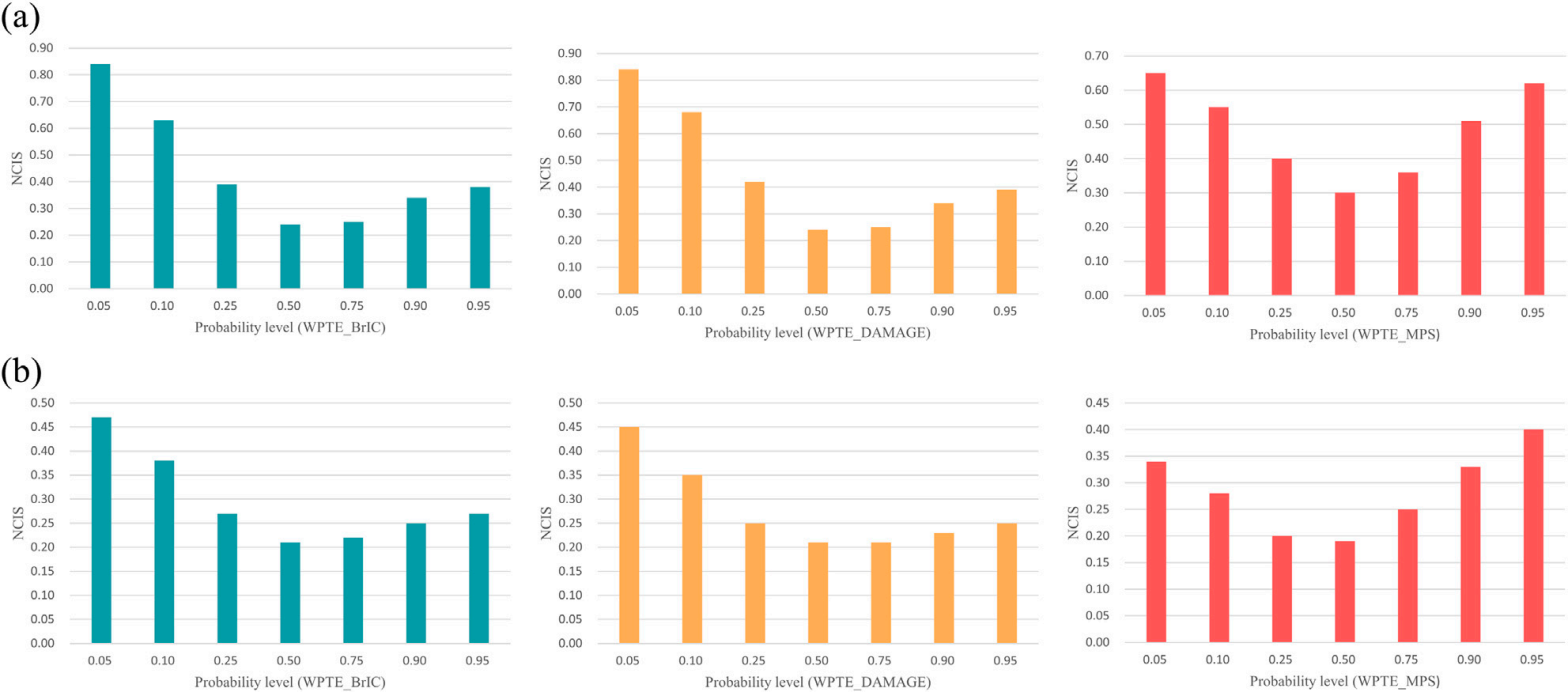
Normalized confidence interval size of IRFs for the optimal distribution: **(a)** rmTBI and **(b)** rsTBI.

**FIGURE 6:**
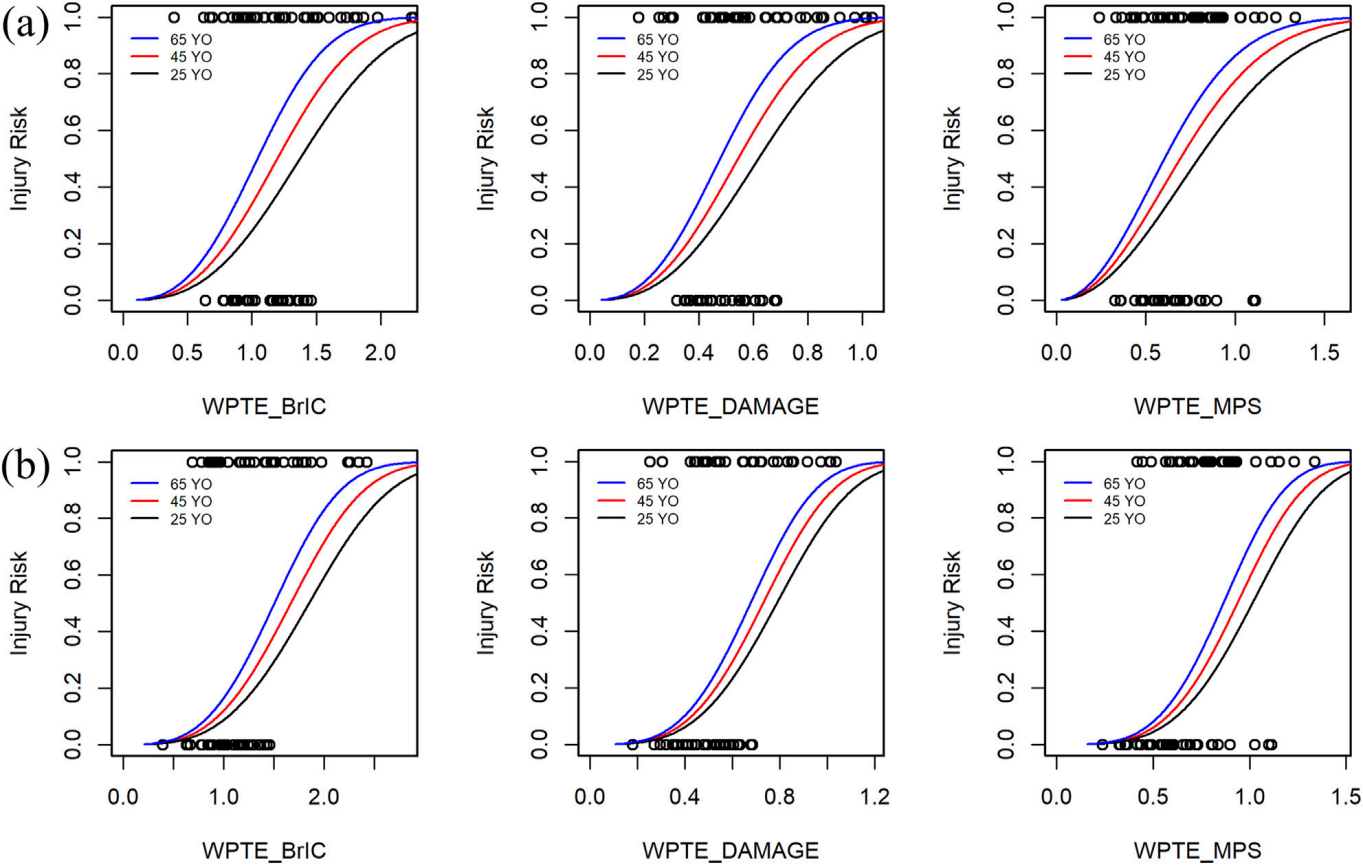
Injury probability of rTBI metrics as a function of predictor variables and separated by three age levels. Data points by censoring (circle). Probability of injury = 1 corresponds to injury, and probability of injury = 0 corresponds to no injury: **(a)** rmTBI and **(b)** rsTBI.

Supplementary Tables C1, C2 provide data on other probabilities, including the magnitude of NCIS. Figure 5 presents a bar graph of the optimal distribution of NCIS. In rmTBI, the quality index for seven risk probability levels is ‘fair’ and ‘good,’ while in rsTBI, the QI for all seven risk probability levels is ‘good.’ Table 1 presents the coefficients and quality index of optimal distribution IRFs, with WPTE_DAMAGE being the best measure for this set, and the predicted values of rmTBI and rsTBI 50% injury risk are 0.494 and 0.678, respectively. The second is WPTE_MPS, with predicted 50% injury risk values of 0.604 and 0.838, respectively. The last is WPTE_BrIC, with predicted 50% injury risk values of 1.085 and 1.513, respectively. In addition, as age increases, the required metrics for injury occurrence instead decrease (Figure 6). For Weibull distribution parameter outputs from the survival analysis, see Supplementary Table C3. The formula is given as follows:

$$P\left(\left.rmTBI\right|{\text{WPTE}}_{X},\text{Age}\right)=1-e^{-{\left[\frac{{\text{WPTE}}_{X}}{e^{\beta_{0}+\beta_{1}A_{\text{ge}}}}\right]}^{\lambda }},$$ (10)

$$P\left(\left.rsTBI\right|{\text{WPTE}}_{X},\text{Age}\right)=1-e^{-{\left[\frac{{\text{WPTE}}_{X}}{e^{\beta_{0}+\beta_{1}A_{\text{ge}}}}\right]}^{\lambda }},$$ (11)

where ${WPTE}_{X}$ is the repetitive traumatic brain injury predictor variable, *β0* and *β1* are the coefficients of the intercept and age covariate, and λ is 1/(scale parameter).

**TABLE 1:** Model coefficient and quality index based on optimal distribution IRFs.

Injury	Metric	Scale (α,μ)	Shape (β)	5% Risk (QI)	25% Risk (QI)	50% Risk (QI)	AIC	Optimal distribution
rmTBI	WPTE_BrIC	1.231	2.89	0.441 (0.83)	0.8 (0.39)	1.085 (0.24)	85.4	Weibull
	WPTE_DAMAGE	0.564	2.778	0.194 (0.86)	0.36 (0.41)	0.494 (0.25)	67.55	Weibull
	WPTE_MPS	0.508	−0.504	0.262 (0.67)	0.429 (0.39)	0.604 (0.30)	75.55	Log-normal
rsTBI	WPTE_BrIC	1.688	3.349	0.695 (0.48)	1.164 (0.27)	1.513 (0.21)	80.19	Weibull
	WPTE_DAMAGE	0.75	3.602	0.329 (0.45)	0.531 (0.25)	0.678 (0.20)	39.3	Weibull
	WPTE_MPS	0.178	−0.177	0.496 (0.34)	0.689 (0.20)	0.838 (0.19)	46.95	Log-logistic

QI: based on the study by Petitjean and Trosseille. (2011), we can categorize the quality index of IRFs into four types, namely, ‘good’ (0–0.5); ‘fair’ (0.5–1.0); ‘marginal' (1.0–1.5); ‘unacceptable’ (>1.5).

## 4 Discussion

In this study, we developed a new approach utilizing pedestrian collision data and WPT to establish metrics for assessing rTBI. The uniqueness of this study, compared to existing brain injury criteria (2019; Prasad and Mertz, 1985 Takhounts et al., 2013; Gabler et al., 2018), lies in simultaneously considering both temporal and frequency-domain features of 6DOF head kinematics, establishing a model for assessing rTBI. By assuming that both time- and frequency-domain information of head kinematics are equally important for brain injury assessment (Laksari et al., 2015; Laksari et al., 2018), we overcome the limitations of evaluating brain injury solely from a time-domain perspective, ensuring a more comprehensive assessment of brain injury through the integration of time-frequency analysis. Finally, three CDFs were used in survival analysis to establish risk curves for rTBI tolerance assessment. We examined goodness-of-fit measures through multiple approaches to establish reliable and well-fitted IRFs.

Many previous studies have evaluated the correlation between different kinematics-based metrics and MPS. HIC and BrIC are based on empirical formulas rather than fundamental mechanical principles. In most studies, HIC has a low goodness-of-fit (low *R* ^2^, Östh et al., 2023), which is expected since it is mainly used to predict skull fracture injuries (Prasad and Mertz, 1985). Although BrIC has a relatively high goodness-of-fit with MPS to some extent, it only relies on peak head kinematics (Takhounts et al., 2013; Prasad et al., 2024a) and cannot account for all possible brain injury scenarios (Takhounts et al., 2013; Prasad et al., 2024b); it is only associated with specific types, simplifying the description of brain injury events. However, many recent research reports have found a high correlation between DAMAGE, constructed based on second-order systems, and MPS (Gabler et al., 2019; Östh et al., 2023; Prasad et al., 2024a; Wu et al., 2022). DAMAGE considers the overall complexity of head impact events in space and time and predicts regional brain injury responses, ultimately assessing potential risks of brain injury (Gabler et al., 2019). The results of this study indirectly indicate that DAMAGE is the best-fitting metric for MPS (Figure 3) and shows better predictive ability (Figure 4), followed by BrIC, while HIC performs poorly. Compared with other rTBI metrics established by combining frequency characteristics, DAMAGE is still the most effective index for quickly assessing the severity of head impact and predicting brain deformation.

Among the rTBI metrics that combine frequency- and time-domain characteristics of head kinematics, the DAMAGE- and MPS-based rTBI metrics showed the best fit in parametric survival analysis (Figure 4) and demonstrated optimal distribution results (Supplementary Figure C1). Strain-based tissue metrics such as MPS and CSDM are considered the most effective metrics for predicting diffuse brain injury, including concussion and diffuse axonal injury (DAI). They reflect the relationship between the strain experienced by the human brain during impact and brain tissue injury (Gennarelli et al., 1972; Takhounts et al., 2013; Alshareef et al., 2020). Studies in various animal models further confirm that head injuries primarily result in diffuse axonal injury, and repeated impacts can also cause various neurological injuries, such as traumatic vascular rupture, increased neuronal excitability, and axonal injury (Fehily and Fitzgerald, 2017; Daneshvar et al., 2023). These findings underscore the critical role of strain in brain tissue injury, further supporting the potential effectiveness of strain-based injury metrics in assessing repetitive brain injuries. Additionally, frequency-based scaling methods demonstrate greater superiority compared to scaling methods based on mass or inertia ratios under biomechanical equivalent loading conditions (Wu et al., 2020). Frequency loading methods can more accurately interpret human responses to mechanical trauma, and combining the frequency domain characteristics with the strain-based metrics (DAMAGE and MPS) can establish a potentially optimal method for assessing rTBI tolerance through survival analysis.

**FIGURE 7:**
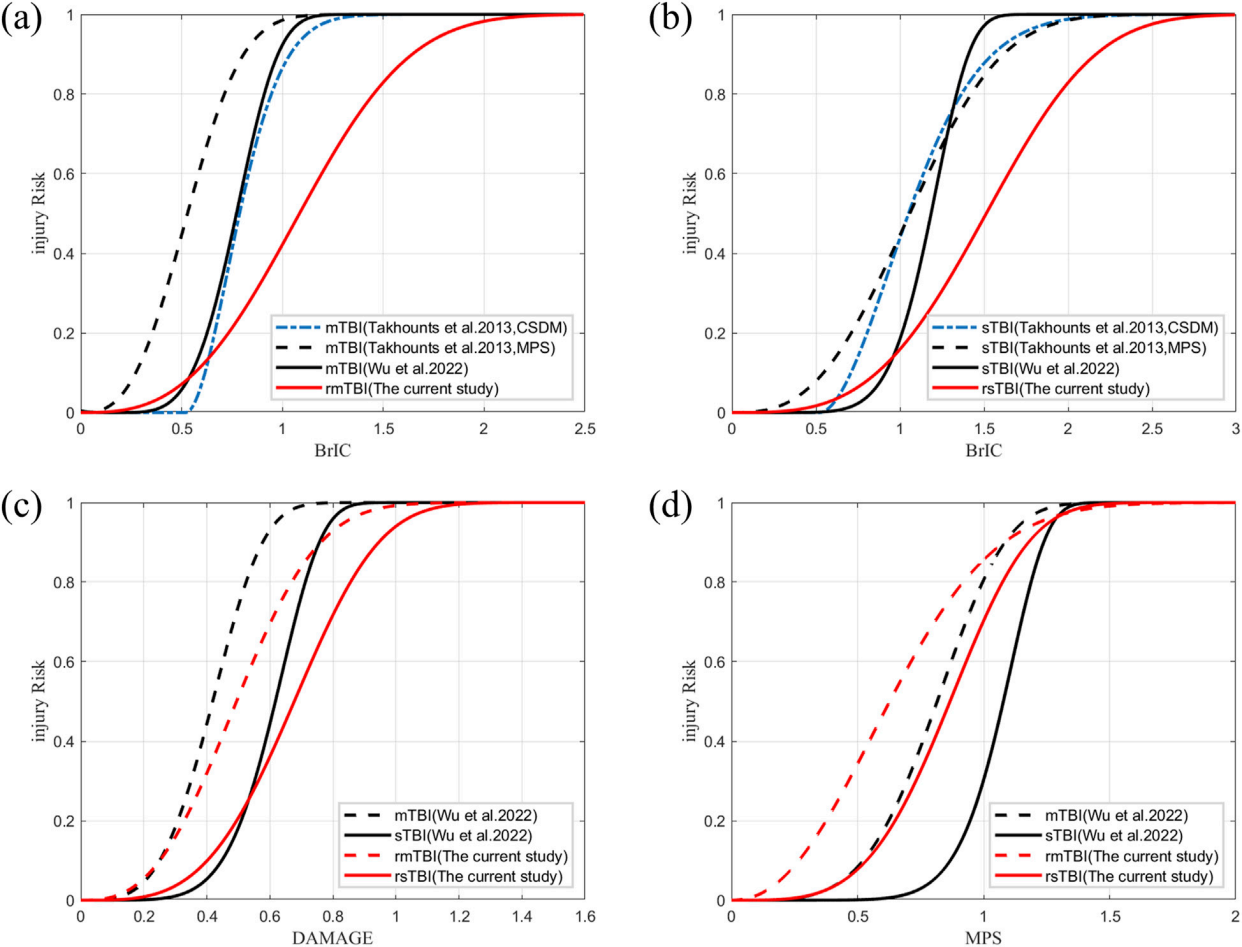
Comparison between developed IRFs and those proposed in the literature. **(a)** Risk curve for mild brain injury based on BrIC. **(b)** Risk curve for severe brain injury based on BrIC. **(c)** Risk curves for mild and severe brain injuries based on DAMAGE. **(d)** Risk curves for mild and severe brain injuries based on MPS.

In exploring the relationship between injury prediction factors and injury risk through reconstructing real-world accidents, many studies have primarily used simple logistic regression methods (Prasad and Mertz, 1985; Peng et al., 2014; Li et al., 2021). However, it is necessary to employ survival analysis for more detailed injury severity and risk analysis. Takhounts et al. (2013) derived two risk curves for BrIC from CSDM and MPS risk curves based on the linear relationship between CSDM-BrIC and MPS-BrIC in animal data, although the IRFs were a poor predictor (Wu et al., 2020; Prasad et al., 2024a). Wu et al. (2022) applied survival analysis to human and animal data to establish IRFs for BrIC, DAMAGE, and MPS, which enhanced our understanding of human brain injury tolerance and was applied in US-NCAP and Euro-NCAP to evaluate the performance of cars. It is undeniable that there are significant differences in the mechanisms of brain injury caused by a single impact *versus* repeated impacts. Existing brain injury standards also show discrepancies compared to the assessment results of repeated brain injury measurements (Figure 3C). However, the injury risk curve established through parametric survival analysis (Figure 7) reveals that, despite the mechanistic differences between single and repeated impacts, there are still certain commonalities in the trend of injury risk growth for both single and repeated impacts.

Standardizing these trends poses challenges due to variations in the types of raw data used to establish IRFs for existing brain injury criteria. In the IRFs of rmTBI (Figure 7A) and rsTBI (Figure 7B) constructed using BrIC, the 50% thresholds of 1.085 and 1.513 (Table 1) for head injury risk are much higher than those determined by Takhounts et al. (2013) based on CSDM thresholds of 0.788 and 1.051, MPS thresholds of 0.529 and 1.059, and Wu et al.’s (2022) thresholds of 0.772 and 1.185. According to reports, the IRF established by the BrIC mass scaling method may not be applicable (Wu et al., 2020) because lower thresholds often substantially overpredict brain injuries caused by repeated impacts in real-world scenarios (Prasad et al., 2024b). For rTBI IRF based on DAMAGE (Figure 7C), the 50% thresholds of 0.494 and 0.678 (Table 1) for head injury risk are slightly higher than those predicted by Wu et al. (2022), which are 0.359 and 0.617; the predicted risks of existing threshold brain injuries are too high compared to real-world experience (Prasad et al., 2024a). However, differences in data censored in survival analysis must be acknowledged. Wu et al. (2022) classified the injury outcome as 1 (left-censored, injury) or 0 (right-censored, no injury). This study used three outcomes for censoring states (left-censored, right-censored, and uncensored). Differences in the classification of data censored can affect the results (Petitjean et al., 2009; Petitjean and Trosseille, 2011). Differences in diagnostic methods or AIS scoring schemes and underestimation of injury risk in early NASS-CDS database entries also influenced the results (Antona-Makoshi et al., 2018). The severity assessment of injuries in kinematic data is relatively conservative compared to the complexity and uncertainty involved in real-world traffic accidents with repetitive collisions and injury risks (Shang et al., 2020). Moreover, there is still a lack of rTBI and biomechanical data to support these efforts.

For the rTBI Weibull distribution IRFs based on MPS (Figure 7D), the thresholds 0.604 and 0.838 (Table 1), as well as the thresholds 0.628 and 0.867 under the optimal distribution (Supplementary Table C4), are lower than the thresholds 0.732 and 1.078 reported by Wu et al. (2022). Consistent with the findings of Stemper et al. (2019), cumulative injury from consecutive head impacts reduces head tolerance and increases the risk of injury. Furthermore, age covariates should be considered for repetitive brain injuries. With increasing age, there are variations in the risk of a given rTBI (Figure 6). Although further validation through *postmortem* human subject experiments is needed, younger specimens require greater impact conditions to result in injuries than older specimens. In short, the IRF of the proposed WPTE_MPS can be used to predict the risk of repetitive brain injury of a human surrogate in a motor vehicle crash. With some necessary optimizations, IRFs have the potential to guide vehicle-to-pedestrian safety research.

There are several limitations to this study. First, only 72 cases of traffic accidents were used, which cannot account for all injury situations in repetitive collisions. Additionally, the study only utilized the THUMS AM50 head model and did not consider the actual age and gender differences in brain tissue, nor did it adequately analyze the effects on the neck and other body parts (Wu et al., 2020). Due to the complexity of rTBI mechanisms and their cumulative effects on head injury tolerance, further exploration is necessary to understand the pathological mechanism of brain injuries in relation to biomechanics. This will help develop more precise and effective protective measures to reduce both the occurrence rate and severity of brain injuries in traffic accidents.

## 5 Conclusion

This study is the first to combine real-world pedestrian collision data and WPT techniques to develop a new method that combines time–frequency domain features of head kinematics and biomechanical responses to assess human tolerance to TBI. The findings are summarized as follows:

1) WPTE_MPS based on tissue-level metrics performs the best in distinguishing the injury degree of rmTBI and rsTBI, followed by WPTE_DAMAGE, which shows the best correlation and fitting measures with WPTE_MPS.
2) The risk curves of mild and severe head injury based on kinematic criteria (WPTE_BrIC and WPTE_DAMAGE) and histological criteria (WPTE_MPS) were established. The 50% probability thresholds for mild and severe head injury were 1.085 and 1.513, 0.494 and 0.678, and 0.628 and 0.838, respectively. In addition, the smaller the index required for injury to occur with increasing age, the more the age covariate should be considered.
3) Standardizing these trends is challenging due to the differences in the original data types used to establish IRFs. The threshold of the rTBI metric based on BrIC and DAMAGE is higher than that of a single collision, indicating that the existing literature may overpredict the risk of brain injury in pedestrian collisions. However, the threshold of the rTBI metric based on MPS is lower than that of a single collision, which implies that the prediction of tolerance to repetitive brain injury is more consistent with tissue-level than head kinematics level, and the existing literature may underestimate the risk of brain injury in pedestrian collisions.

## Data Availability

The original contributions presented in the study are included in the article/Supplementary Material; further inquiries can be directed to the corresponding authors.

## References

[B1] Alshareef A. Giudice J. S. Forman J. Salzar R. S. Panzer M. B. (2018). A novel method for quantifying human *in situ* whole brain deformation under rotational loading using sonomicrometry. J. Neurotrauma 35 (5), 780–789. 10.1089/neu.2017.5362 29179620

[B2] Alshareef A. Giudice J. S. Forman J. Shedd D. F. Reynier K. A. Wu T. (2020). Biomechanics of the human brain during dynamic rotation of the head. J. Neurotrauma 37 (13), 1546–1555. 10.1089/neu.2019.6847 31952465 PMC7307677

[B3] Antona-Makoshi J. Mikami K. Lindkvist M. Davidsson J. Schick S. (2018). Accident analysis to support the development of strategies for the prevention of brain injuries in car crashes. Accid. Anal. Prev. 117, 98–105. 10.1016/j.aap.2018.04.009 29679852

[B4] Daneshvar D. H. Nair E. S. Baucom Z. H. Rasch A. Abdolmohammadi B. Uretsky M. (2023). Leveraging football accelerometer data to quantify associations between repetitive head impacts and chronic traumatic encephalopathy in males. Nat. Commun. 14 (1), 3470. 10.1038/s41467-023-39183-0 37340004 PMC10281995

[B5] Di Domenico L. Nusholtz G. (2005). Risk curve boundaries. Traffic Inj. Prev. 6 (1), 86–94. 10.1080/15389580590903212 15823880

[B6] Fehily B. Fitzgerald M. (2017). Repeated mild traumatic brain injury: potential mechanisms of damage. Cell Transpl. 26 (7), 1131–1155. 10.1177/0963689717714092 PMC5657727 28933213

[B7] Gabler L. F. Crandall J. R. Panzer M. B. (2018). Development of a metric for predicting brain strain responses using head kinematics. Ann. Biomed. Eng. 46 (7), 972–985. 10.1007/s10439-018-2015-9 29594689

[B8] Gabler L. F. Crandall J. R. Panzer M. B. (2019). Development of a second-order system for rapid estimation of maximum brain strain. Ann. Biomed. Eng. 47 (9), 1971–1981. 10.1007/s10439-018-02179-9 30515603

[B9] Gennarelli T. A. Thibault L. E. Ommaya A. K. (1972). Pathophysiologic responses to rotational and translational accelerations of the head (No. 720970). SAE Tech. Pap.

[B10] Guillaume A. Hermitte T. Hervé V. Fricheteau R. (2015). “Car or ground: which causes more pedestrian injuries?,” in 24th international technical conference on the enhanced safety of vehicles (ESV) national highway traffic safety administration, 15–0084.

[B11] Gurdjian E. S. Roberts V. L. Thomas L. M. (1966). Tolerance curves of acceleration and intracranial pressure and protective index in experimental head injury. J. Trauma 6 (5), 600–604. 10.1097/00005373-196609000-00005 5928630

[B12] Iwamoto M. Nakahira Y. Kimpara H. (2015). Development and validation of the total human model for safety (THUMS) toward further understanding of occupant injury mechanisms in precrash and during crash. Traffic Inj. Prev. 16 (Suppl. 1), S36–S48. 10.1080/15389588.2015.1015000 26027974

[B13] Jean A. Nyein M. K. Zheng J. Q. Moore D. F. Joannopoulos J. D. Radovitzky R. (2014). An animal-to-human scaling law for blast-induced traumatic brain injury risk assessment. Proc. Natl. Acad. Sci. U. S. A. 111 (43), 15310–15315. 10.1073/pnas.1415743111 25267617 PMC4217421

[B14] Kanianthra J. N. Fan W. Rains G. (1996). Upper interior head impact protection of occupants in real world crashes. Proc. Int. Tech. Conf. Enhanc. Saf. Veh. 1996, 1281–1290. National Highway Traffic Safety Administration.

[B15] Kent R. W. Funk J. R. (2004). Data censoring and parametric distribution assignment in the development of injury risk functions from biochemical data (No. 2004-01-0317). SAE Technical Paper.

[B16] Laksari K. Kurt M. Babaee H. Kleiven S. Camarillo D. (2018). Mechanistic insights into human brain impact dynamics through modal analysis. Phys. Rev. Lett. 120 (13), 138101. 10.1103/physrevlett.120.138101 29694192

[B17] Laksari K. Wu L. C. Kurt M. Kuo C. Camarillo D. C. (2015). Resonance of human brain under head acceleration. J. R. Soc. Interface 12 (108), 20150331. 10.1098/rsif.2015.0331 26063824 PMC4528602

[B18] Li G. Liu J. Li K. Zhao H. Shi L. Zhang S. (2021). Realistic reference for evaluation of vehicle safety focusing on pedestrian head protection observed from kinematic reconstruction of real-world collisions. Front. Bioeng. Biotechnol. 9, 768994. 10.3389/fbioe.2021.768994 34993187 PMC8724547

[B19] Li G. Xu S. Xiong T. Li K. Qiu J. (2024). Characteristics of head frequency response in blunt impacts: a biomechanical modeling study. Front. Bioeng. Biotechnol. 12, 1364741. 10.3389/fbioe.2024.1364741 38468687 PMC10925751

[B20] O'Connor K. L. Rowson S. Duma S. M. Broglio S. P. (2017). Head-impact–measurement devices: a systematic review. J. Athl. Train. 52 (3), 206–227. 10.4085/1062-6050.52.2.05 28387553 PMC5384819

[B21] Östh J. Bohman K. Jakobsson L. (2023). Head injury criteria assessment using head kinematics from crash tests and accident reconstructions. Traffic Inj. Prev. 24 (1), 56–61. 10.1080/15389588.2022.2143238 36374230

[B22] Peng Y. Yang J. Deck C. Otte D. Willinger R. (2014). Development of head injury risk functions based on real-world accident reconstruction. Int. J. Crashworthiness. 19 (2), 105–114. 10.1080/13588265.2013.805290

[B23] Petitjean A. Trosseille X. (2011). Statistical simulations to evaluate the methods of the construction of injury risk curves (No. 2011-22-0015). SAE Technical Paper. 10.4271/2011-22-0015 22869316

[B24] Petitjean A. Trosseille X. Petit P. Irwin A. Hassan J. Praxl N. (2009). Injury risk curves for the WorldSID 50th male dummy (No. 2009-22-0016). *SAE* Technical Paper. 10.4271/2009-22-0016 20058564

[B25] Prasad P. Barbat S. D. Kalra A. Dalmotas D. J. (2024b). Evaluation of DAMAGE algorithm in frontal crashes. Stapp Car Crash J. 67 (2023-22-0006), 171–179. 10.4271/2023-22-0006 38662624

[B26] Prasad P. Barbat S. D. Kalra A. Kim A. S. Dalmotas D. D. Zhang L. (2024a). Evaluation of brain rotational injury criteria (BrIC) in vehicle frontal crashes. Traffic Inj. Prev. 25 (1), 57–64. 10.1080/15389588.2023.2255913 37706464

[B27] Prasad P. Mertz H. J. (1985). The position of the United States delegation to the ISO Working Group 6 on the use of HIC in the automotive environment. SAE Trans., 106–116.

[B28] Shang S. Masson C. Teeling D. Py M. Ferrand Q. Arnoux P. J. (2020). Kinematics and dynamics of pedestrian head ground contact: a cadaver study. Saf. Sci. 127, 104684. 10.1016/j.ssci.2020.104684

[B29] Shi L. Han Y. Huang H. Davidsson J. Thomson R. (2020). Evaluation of injury thresholds for predicting severe head injuries in vulnerable road users resulting from ground impact via detailed accident reconstructions. Biomech. Model Mechanobiol. 19, 1845–1863. 10.1007/s10237-020-01312-9 32133546

[B30] Shi L. Han Y. Huang H. Li Q. Wang B. Mizuno K. (2018). Analysis of pedestrian-to-ground impact injury risk in vehicle-to-pedestrian collisions based on rotation angles. J. Saf. Res. 64, 37–47. 10.1016/j.jsr.2017.12.004 29636168

[B31] Stemper B. D. Shah A. S. Harezlak J. Rowson S. Mihalik J. P. Duma S. M. (2019). Comparison of head impact exposure between concussed football athletes and matched controls: evidence for a possible second mechanism of sport-related concussion. Ann. Biomed. Eng. 47 (10), 2057–2072. 10.1007/s10439-018-02136-6 30362082 PMC6785644

[B32] Takhounts E. G. Craig M. J. Moorhouse K. McFadden J. Hasija V. (2013). Development of brain injury criteria (BrIC) (No. 2013-22-0010). SAE Technical Paper. 10.4271/2013-22-0010 24435734

[B33] Wang Z. Wei L. Cao M. (2019). Damage quantification with embedded piezoelectric aggregates based on wavelet packet energy analysis. Sensors 19 (2), 425. 10.3390/s19020425 30669631 PMC6359494

[B34] World Health Organization (2023). Global status report on road safety 2023: summary. World Health Organization.

[B35] Wu T. Antona-Makoshi J. Alshareef A. Giudice J. S. Panzer M. B. (2020). Investigation of cross-species scaling methods for traumatic brain injury using finite element analysis. J. Neurotrauma 37 (2), 410–422. 10.1089/neu.2019.6576 31382861

[B36] Wu T. Hajiaghamemar M. Giudice J. S. Alshareef A. Margulies S. S. Panzer M. B. (2021). Evaluation of tissue-level brain injury metrics using species-specific simulations. J. Neurotrauma 38 (13), 1879–1888. 10.1089/neu.2020.7445 33446011 PMC8219195

[B37] Wu T. Sato F. Antona-Makoshi J. Gabler L. F. Giudice J. S. Alshareef A. (2022). Integrating human and nonhuman primate data to estimate human tolerances for traumatic brain injury. J. Biomech. Eng. 144 (7), 071003. 10.1115/1.4053209 34897386

