# Supplementary material for: Development of a repetitive traumatic brain injury risk function based on real-world accident reconstruction and wavelet packet energy analysis
Source: Front Bioeng Biotechnol. 2025 Mar 26;13:1548265. doi: 10.3389/fbioe.2025.1548265 (PMC11980440; doi:10.3389/fbioe.2025.1548265)
Supplement: Supplementary file 1 [file DataSheet1.docx]

# 1 Appendix A

All accident cases were carefully reconstructed and damage scored in each case by a professional team of accident investigators and medical professionals. In order to make the collected accident cases more effective for the establishment of repetitive collision injury risk function, the following selection criteria were selected, including:

(1) Classification of head injuries based on the 2005 Abbreviated Injury Scale (AIS), ranging from AIS1+ to AIS5+ levels;

(2) Inclusion of only those cases where pedestrians experienced secondary contact with the ground following initial collision with the front portion of a vehicle;

(3) Documentation of information such as vehicle model, age, and description of injury site;

(4) Ability to calculate or estimate pertinent collision details including vehicle impact speed, initial contact position, ground contact position and attitude.

Table A1. Summary of the information for cases reconstructed

| Case No. | Vehicle type | Vehicle (km/h) | Gender | age |
| --- | --- | --- | --- | --- |
| 1 | Sedan | 56 | M | 77 |
| 2 | Sedan | 26 | F | 59 |
| 3 | Sedan | 46 | F | 51 |
| 4 | Sedan | 55 | M | 51 |
| 5 | Sedan | 47 | F | 56 |
| 6 | Sedan | 26 | M | 75 |
| 7 | Sedan | 45 | F | 83 |
| 8 | MPV | 43 | F | 71 |
| 9 | Sedan | 56 | M | 60 |
| 10 | Sedan | 69 | F | 71 |
| 11 | MPV | 37 | M | 61 |
| 12 | Sedan | 39 | M | 32 |
| 13 | MPV | 53 | F | 61 |
| 14 | MPV | 48 | F | 68 |
| 15 | Sedan | 40 | M | 84 |
| 16 | MPV | 46 | M | 29 |
| 17 | MPV | 56 | F | 56 |
| 18 | MPV | 51 | F | 87 |
| 19 | Sedan | 55 | F | 76 |
| 20 | MPV | 44 | M | 63 |
| 21 | Sedan | 55 | M | 65 |
| 22 | MPV | 31 | M | 57 |
| 23 | Sedan | 60 | F | 59 |
| 24 | MPV | 36 | F | 96 |
| 25 | Sedan | 64 | M | 81 |
| 26 | Sedan | 50 | M | 53 |
| 27 | Sedan | 67 | F | 72 |
| 28 | Sedan | 50 | F | 42 |
| 29 | Sedan | 31 | F | 77 |
| 30 | Sedan | 63 | M | 76 |
| 31 | Sedan | 78 | M | 64 |
| 32 | Sedan | 49 | F | 78 |
| 33 | SUV | 24 | F | 83 |
| 34 | sedan | 75 | M | 67 |
| 35 | sedan | 31 | M | 51 |
| 36 | SUV | 65 | F | 31 |
| 37 | sedan | 77 | M | 56 |
| 38 | Sedan | 40 | F | 27 |
| 39 | Sedan | 43 | F | 76 |
| 40 | Sedan | 32 | M | 83 |
| 41 | Sedan | 35 | F | 68 |
| 42 | Sedan | 25 | M | 74 |
| 43 | Sedan | 50 | F | 51 |
| 44 | Sedan | 40 | F | 71 |
| 45 | Sedan | 32 | F | 85 |
| 46 | Sedan | 59 | M | 67 |
| 47 | Sedan | 41 | M | 68 |
| 48 | Sedan | 40 | F | 76 |
| 49 | Sedan | 35 | M | 57 |
| 50 | Sedan | 36 | F | 20 |
| 51 | Sedan | 47 | M | 26 |
| 52 | Sedan | 42 | M | 56 |
| 53 | Sedan | 30 | M | 23 |
| 54 | Sedan | 48 | M | 29 |
| 55 | Sedan | 25 | F | 44 |
| 56 | Sedan | 30 | F | 78 |
| 57 | Sedan | 34 | M | 27 |
| 58 | Sedan | 32 | M | 31 |
| 59 | Sedan | 31 | F | 71 |
| 60 | Sedan | 45 | F | 47 |
| 61 | Sedan | 35 | M | 66 |
| 62 | Sedan | 30 | F | 56 |
| 63 | Sedan | 45 | F | 70 |
| 64 | Sedan | 40 | F | 62 |
| 65 | Sedan | 30 | M | 22 |
| 66 | Sedan | 30 | M | 18 |
| 67 | Sedan | 50 | F | 67 |
| 68 | Sedan | 30 | F | 21 |
| 69 | Sedan | 40 | M | 54 |
| 70 | Sedan | 35 | M | 76 |
| 71 | Sedan | 22 | F | 67 |
| 72 | Sedan | 36 | M | 45 |
|  |  |  |  |  |

Table A2. Summary of Survival analysis data types records and AIS code. (Association for the Advancement Automotive Medicine, 2005)

| Case No. | AIS code | Skull fracture: Yes (1)/No (0) | Death: Yes (1)/No (0) | MAIS | Censoring | |
| --- | --- | --- | --- | --- | --- | --- |
|  |  |  |  |  | rmTBI(AIS2-3) | rsTBI(AIS4-5) |
| 1 | 110099.1,140610.5 ,150204.3 ,140629.3 ,120099.3 | 1 | 1 | 5 | Left | Uncensored |
| 2 | 140650.3 ,140402.3,140610.5 ,140695.3 ,150204.3,140682.3 ,110099.1 ,251222.2 ,251006.2 | 1 | 1 | 5 | Left | Uncensored |
| 3 | 140404.4 ,140610.5,140666.5,140204.5 ,140695.3,140650.3 ,150000.2 ,110099.1 ,251900.3 | 1 | 1 | 5 | Left | Uncensored |
| 4 | 140650.3,140628.4 ,140210.5 ,140666.5,140695.3 ,140616.4 ,140682.3 ,150200.3 | 1 | 1 | 5 | Left | Uncensored |
| 5 | 140611.3,140693.2 ,150000.2 ,110099.1 | 1 | 0 | 3 | Uncensored | Right |
| 6 | 140624.4 ,140695.3,140656.5 ,140660.3,150200.3 ,110099.1 | 1 | 1 | 5 | Left | Uncensored |
| 7 | 110604.2 ,140611.3 ,140628.4,110402.1 | 0 | 1 | 4 | Left | Uncensored |
| 8 | 161013.5,110600.1,113000.6 | 0 | 1 | 6 | Left | Left |
| 9 | 140693.2 ,120099.3,110600.1 ,140608.4,140660.3 | 0 | 1 | 4 | Left | Uncensored |
| 10 | 140204.5,140602.3 ,140693.2 ,140631.2 ,110099.1 | 0 | 1 | 5 | Left | Uncensored |
| 11 | 140629.3 ,150204.3 ,140699.3,113000.6 | 1 | 1 | 6 | Left | Left |
| 12 | 140650.3 ,140693.2 ,150200.3,150000.2 ,140682.3,110600.1 | 1 | 0 | 3 | Uncensored | Right |
| 13 | 140693.2,110099.1 | 0 | 0 | 2 | Uncensored | Right |
| 14 | 140204.5 ,140628.4 ,140602.3,150200.3 ,110099.1 | 1 | 0 | 5 | Left | Uncensored |
| 15 | 150204.3,140650.3 ,140695.3 ,140682.3 ,140666.5 ,251211.2 | 1 | 1 | 5 | Left | Uncensored |
| 16 | 110604.2,110202.1,140652.4 ,120099.3 ,150200.3 ,140674.5 ,140693.2 ,113000.6 | 1 | 1 | 6 | Left | Left |
| 17 | 150204.3,110099.1 ,140629.3,140652.4 | 1 | 1 | 4 | Left | Uncensored |
| 18 | 140604.3 ,140650.3 ,140693.2 ,150200.3,110099.1,161013.5 | 1 | 1 | 5 | Left | Uncensored |
| 19 | 140652.4 ,140602.3 ,140664.4,150000.2,150200.3 ,140204.5,110099.1 ,113000.6 | 1 | 1 | 6 | Left | Left |
| 20 | 140650.3 ,140693.2 ,110202.1 ,251800.1 ,210402.1,140628.4 | 1 | 0 | 4 | Left | Uncensored |
| 21 | 140602.3,140650.3 ,140693.2 ,150000.2 ,251800.1 ,251200.2,140628.4 | 1 | 0 | 4 | Left | Uncensored |
| 22 | 110600.1 ,150204.3 ,120099.3 ,113000.6 | 1 | 1 | 6 | Left | Left |
| 23 | 140602.3 ,140650.3 ,110099.1, 140674.5 | 0 | 1 | 5 | Left | Uncensored |
| 24 | 110604.2 ,150206.4 ,140614.3 ,140629.3 | 1 | 1 | 4 | Left | Uncensored |
| 25 | 140602.3,140650.3 ,140693.2 ,150400.2,140628.4 | 1 | 0 | 4 | Left | Uncensored |
| 26 | 140602.3,140629.3,140650.3 ,140693.2 ,150000.2 ,150200.3 ,140628.4 | 1 | 0 | 4 | Left | Uncensored |
| 27 | 110604.2,150000.2 ,140602.3 ,140695.3 ,161011.5 ,113000.6 | 1 | 1 | 6 | Left | Left |
| 28 | 251200.2 ,161002.2 | 0 | 0 | 2 | Uncensored | Right |
| 29 | 110202.1,161000.1 | 0 | 0 | 1 | Right | Right |
| 30 | 150200.3,140204.5 ,140602.3 | 1 | 1 | 5 | Left | Uncensored |
| 31 | 150200.3 ,150400.2 ,161013.5 ,110099.1 | 1 | 1 | 5 | Left | Uncensored |
| 32 | 140693.2 ,150000.2,130899.2 ,110099.1,113000.6 | 1 | 0 | 6 | Left | Left |
| 33 | 110099.1 ,140693.2 ,140611.3 ,150000.2 ,140650.3 ,113000.6 | 1 | 0 | 6 | Left | Left |
| 34 | 150400.2,110099.1 ,140499.3,140602.3 ,140650.3 ,140628.4 | 1 | 1 | 4 | Left | Uncensored |
| 35 | 140602.3 ,140650.3,140693.2 ,110099.1,251408.1 ,140666.5 | 0 | 0 | 5 | Left | Uncensored |
| 36 | 110099.1,140611.3 ,140666.5 ,161011.5 ,150200.3 ,140695.3,113000.6 | 1 | 1 | 6 | Left | Left |
| 37 | 140666.5 ,140629.3 ,150200.3,161013.5 ,140650.3 ,110099.1 ,113000.6 | 1 | 1 | 6 | Left | Left |
| 38 | 110202.1 | 0 | 0 | 1 | Right | Right |
| 39 | 110402.1 | 0 | 0 | 1 | Right | Right |
| 40 | 110602.1 | 0 | 0 | 1 | Right | Right |
| 41 | 110602.1 | 0 | 0 | 1 | Right | Right |
| 42 | 110602.1 | 0 | 0 | 1 | Right | Right |
| 43 | 110602.1，140684.3，150202.3，150200.3 | 1 | 0 | 3 | Uncensored | Right |
| 44 | 110602.1 | 0 | 0 | 1 | Right | Right |
| 45 | 110202.1 | 0 | 0 | 1 | Right | Right |
| 46 | 110202.1 | 0 | 0 | 1 | Right | Right |
| 47 | 110402.1 | 0 | 0 | 1 | Right | Right |
| 48 | 140648.5，150200.3 | 1 | 0 | 5 | Left | Uncensored |
| 49 | 140602.3，150200.3，150404.3 | 1 | 0 | 3 | Uncensored | Right |
| 50 | 110402.1 | 0 | 0 | 1 | Right | Right |
| 51 | 110402.1 | 0 | 0 | 1 | Right | Right |
| 52 | 110202.1 | 0 | 0 | 1 | Right | Right |
| 53 | 110202.1 | 0 | 0 | 1 | Right | Right |
| 54 | 110402.1 | 0 | 0 | 1 | Right | Right |
| 55 | 110402.1 | 0 | 0 | 1 | Right | Right |
| 56 | 110602.1 | 0 | 0 | 1 | Right | Right |
| 57 | 110402.1 | 0 | 0 | 1 | Right | Right |
| 58 | 110402.1 | 0 | 0 | 1 | Right | Right |
| 59 | 110602.1 | 0 | 0 | 1 | Right | Right |
| 60 | 161000.2 | 0 | 0 | 2 | Uncensored | Right |
| 61 | 110202.1 | 0 | 0 | 1 | Right | Right |
| 62 | 110202.1 | 0 | 0 | 1 | Right | Right |
| 63 | 161000.2 | 0 | 0 | 2 | Uncensored | Right |
| 64 | 161000.2 | 0 | 0 | 2 | Uncensored | Right |
| 65 | 110402.1 | 0 | 0 | 1 | Right | Right |
| 66 | 110202.1 | 0 | 0 | 1 | Right | Right |
| 67 | 110602.1 | 0 | 0 | 1 | Right | Right |
| 68 | 110402.1 | 0 | 0 | 1 | Right | Right |
| 69 | 110602.1，140699.3，150200.3 | 1 | 0 | 3 | Uncensored | Right |
| 70 | 161000.2 | 0 | 0 | 2 | Uncensored | Right |
| 71 | 161000.2 | 0 | 0 | 2 | Uncensored | Right |
| 72 | 110600.1 | 0 | 0 | 1 | Right | Right |

Table A3. Summary of calculated head injury parameters for cases reconstructed

|  | **vehicle contact** | | | | | | |  | **ground contact** | | | | | | |
| --- | --- | --- | --- | --- | --- | --- | --- | --- | --- | --- | --- | --- | --- | --- | --- |
|  | **Head kinematic-based criteria** | | | | |  | **Tissue-level injury criteria** |  | **Head kinematic-based criteria** | | | | |  | **Tissue-level injury criteria** |
| **Case No.** | **HIC** | **BrIC** | **DAMAGE** | **Energy_RLA** | **Energy_RAA** |  | **MPS** |  | **HIC** | **BrIC** | **DAMAGE** | **Energy_RLA** | **Energy_RAA** |  | **MPS** |
| 1 | 2827.66 | 1.38 | 0.63 | 4.09E+08 | 8.47E+09 |  | 0.3196 |  | 4584.42 | 1.19 | 0.422 | 3.74E+08 | 3.56E+10 |  | 0.7291 |
| 2 | 984.88 | 0.53 | 0.234 | 1.37E+08 | 2.76E+09 |  | 0.3536 |  | 51.17 | 0.47 | 0.273 | 1.08E+07 | 9.70E+09 |  | 0.4526 |
| 3 | 67 | 0.52 | 0.267 | 2.45E+07 | 1.89E+09 |  | 0.528 |  | 5107.4 | 0.63 | 0.332 | 3.06E+08 | 9.08E+09 |  | 0.4771 |
| 4 | 1991.72 | 0.57 | 0.345 | 2.68E+08 | 3.97E+09 |  | 0.2593 |  | 3712.78 | 0.79 | 0.248 | 2.97E+08 | 1.72E+10 |  | 0.5459 |
| 5 | 452.92 | 0.38 | 0.203 | 7.38E+07 | 1.33E+09 |  | 0.1814 |  | 758.03 | 0.34 | 0.127 | 1.17E+08 | 3.50E+09 |  | 0.3225 |
| 6 | 225.67 | 0.4 | 0.153 | 4.32E+07 | 8.80E+08 |  | 0.1596 |  | 1886.84 | 0.36 | 0.121 | 2.78E+08 | 3.99E+09 |  | 0.4032 |
| 7 | 351.65 | 0.93 | 0.346 | 8.18E+07 | 3.80E+09 |  | 0.5184 |  | 1052.4 | 0.59 | 0.173 | 1.69E+08 | 4.68E+09 |  | 0.5159 |
| 8 | 524.05 | 0.85 | 0.314 | 1.49E+08 | 2.91E+09 |  | 0.4982 |  | 1719 | 0.52 | 0.283 | 1.69E+08 | 4.25E+09 |  | 0.4515 |
| 9 | 973.97 | 0.67 | 0.344 | 1.75E+08 | 5.55E+09 |  | 0.2734 |  | 4308.2 | 0.66 | 0.191 | 3.21E+08 | 1.63E+10 |  | 0.3912 |
| 10 | 1347 | 1.17 | 0.389 | 1.88E+08 | 8.89E+09 |  | 0.556 |  | 19801 | 0.83 | 0.211 | 1.07E+09 | 2.63E+10 |  | 0.4171 |
| 11 | 1087.1 | 0.68 | 0.325 | 1.23E+08 | 3.42E+09 |  | 0.1762 |  | 1167.3 | 0.44 | 0.307 | 2.01E+08 | 4.23E+09 |  | 0.7405 |
| 12 | 524.9 | 0.65 | 0.244 | 1.39E+08 | 4.14E+09 |  | 0.4284 |  | 3032.75 | 0.82 | 0.276 | 2.88E+08 | 1.06E+10 |  | 0.5274 |
| 13 | 798.26 | 0.63 | 0.254 | 1.02E+08 | 1.83E+09 |  | 0.2773 |  | 13.11 | 0.25 | 0.109 | 7.71E+06 | 2.90E+08 |  | 0.3904 |
| 14 | 2271 | 0.59 | 0.361 | 3.02E+08 | 3.75E+09 |  | 0.3386 |  | 4619.64 | 0.41 | 0.137 | 3.27E+08 | 6.70E+09 |  | 0.4523 |
| 15 | 1409.4 | 0.72 | 0.285 | 2.07E+08 | 2.58E+09 |  | 0.3795 |  | 4432.3 | 0.82 | 0.244 | 3.34E+08 | 1.30E+10 |  | 0.6202 |
| 16 | 3244 | 1.1 | 0.556 | 3.60E+08 | 1.02E+10 |  | 0.3996 |  | 7467.9 | 0.85 | 0.37 | 5.74E+08 | 3.00E+10 |  | 0.698 |
| 17 | 4008.2 | 1.15 | 0.547 | 4.40E+08 | 7.49E+09 |  | 0.6302 |  | 8789.6 | 1.42 | 0.543 | 4.55E+08 | 6.66E+10 |  | 0.535 |
| 18 | 4995.16 | 0.87 | 0.482 | 4.30E+08 | 8.17E+09 |  | 0.2639 |  | 6859.96 | 0.84 | 0.379 | 5.59E+08 | 2.67E+10 |  | 0.6618 |
| 19 | 3831.8 | 0.77 | 0.4 | 4.33E+08 | 4.56E+09 |  | 0.4262 |  | 26817 | 1.09 | 0.332 | 1.71E+09 | 2.56E+10 |  | 0.5453 |
| 20 | 1255.8 | 0.79 | 0.32 | 1.81E+08 | 3.47E+09 |  | 0.2534 |  | 4192.6 | 0.85 | 0.295 | 4.28E+08 | 1.90E+10 |  | 0.6029 |
| 21 | 2529.88 | 0.65 | 0.443 | 4.06E+08 | 6.94E+09 |  | 0.5203 |  | 528.13 | 0.48 | 0.178 | 1.18E+08 | 6.40E+09 |  | 0.5367 |
| 22 | 623.83 | 0.48 | 0.262 | 8.92E+07 | 1.70E+09 |  | 0.2779 |  | 7550.3 | 0.89 | 0.414 | 2.33E+08 | 2.21E+10 |  | 0.6199 |
| 23 | 1344.46 | 0.91 | 0.454 | 1.60E+08 | 6.36E+09 |  | 0.2938 |  | 3581.5 | 0.78 | 0.323 | 2.86E+08 | 1.71E+10 |  | 0.8228 |
| 24 | 4199.98 | 0.78 | 0.439 | 3.52E+08 | 6.53E+09 |  | 0.2276 |  | 5818.3 | 0.81 | 0.35 | 4.86E+08 | 2.56E+10 |  | 0.7049 |
| 25 | 5549.87 | 0.86 | 0.512 | 4.93E+08 | 8.44E+09 |  | 0.5325 |  | 3239.1 | 0.59 | 0.244 | 2.71E+08 | 9.73E+09 |  | 0.2889 |
| 26 | 2557 | 0.61 | 0.367 | 2.91E+08 | 3.87E+09 |  | 0.3245 |  | 6629.2 | 0.49 | 0.237 | 4.88E+08 | 8.39E+09 |  | 0.4693 |
| 27 | 5610.7 | 0.94 | 0.584 | 5.60E+08 | 1.08E+10 |  | 0.3758 |  | 10967 | 0.74 | 0.371 | 7.60E+08 | 3.00E+10 |  | 0.5726 |
| 28 | 1077.9 | 0.33 | 0.144 | 1.34E+08 | 7.39E+08 |  | 0.2378 |  | 71.01 | 0.16 | 0.086 | 1.52E+07 | 4.87E+08 |  | 0.324 |
| 29 | 16.93 | 0.42 | 0.145 | 1.65E+07 | 4.51E+08 |  | 0.14 |  | 5750.4 | 0.58 | 0.214 | 4.78E+08 | 7.83E+09 |  | 0.3138 |
| 30 | 5943.9 | 1.22 | 0.561 | 6.36E+08 | 1.22E+10 |  | 0.3054 |  | 11543 | 1.08 | 0.407 | 6.15E+08 | 2.94E+10 |  | 0.7833 |
| 31 | 16119 | 1.01 | 0.657 | 1.34E+09 | 1.29E+10 |  | 0.825 |  | 21218 | 1.13 | 0.465 | 1.18E+09 | 4.02E+10 |  | 0.6761 |
| 32 | 1724.9 | 0.7 | 0.324 | 2.61E+08 | 3.72E+09 |  | 0.2576 |  | 199.96 | 0.61 | 0.221 | 6.01E+07 | 2.99E+09 |  | 0.4199 |
| 33 | 96.36 | 0.28 | 0.117 | 2.41E+07 | 3.97E+08 |  | 0.1039 |  | 3528.5 | 0.52 | 0.196 | 2.72E+08 | 8.91E+09 |  | 0.5137 |
| 34 | 8422.9 | 1 | 0.513 | 8.23E+08 | 1.14E+10 |  | 0.4224 |  | 22963 | 1.47 | 0.586 | 1.02E+09 | 6.65E+10 |  | 0.9445 |
| 35 | 808.94 | 1.13 | 0.345 | 2.87E+08 | 3.49E+09 |  | 0.3226 |  | 1957.8 | 1.23 | 0.418 | 1.77E+08 | 3.16E+10 |  | 0.7868 |
| 36 | 4219.7 | 1.06 | 0.561 | 5.25E+08 | 8.62E+09 |  | 0.3037 |  | 6322.9 | 0.93 | 0.433 | 4.01E+08 | 3.84E+10 |  | 0.7686 |
| 37 | 7898.1 | 0.79 | 0.445 | 8.65E+08 | 7.95E+09 |  | 0.4626 |  | 3626.1 | 1.03 | 0.437 | 4.33E+08 | 3.00E+10 |  | 0.8722 |
| 38 | 1844.2 | 0.68 | 0.304 | 2.68E+08 | 3.57E+09 |  | 0.3277 |  | 4228.4 | 0.79 | 0.318 | 3.95E+08 | 1.21E+10 |  | 0.4435 |
| 39 | 1446.1 | 0.65 | 0.407 | 2.22E+08 | 3.11E+09 |  | 0.2931 |  | 17544 | 0.83 | 0.322 | 9.56E+08 | 1.88E+10 |  | 0.941 |
| 40 | 1702.8 | 0.55 | 0.344 | 1.86E+08 | 2.67E+09 |  | 0.2242 |  | 4915.3 | 0.88 | 0.367 | 3.96E+08 | 2.58E+10 |  | 0.7416 |
| 41 | 1022.1 | 0.55 | 0.324 | 1.69E+08 | 2.15E+09 |  | 0.242 |  | 1164.8 | 0.78 | 0.272 | 1.88E+08 | 9.64E+09 |  | 0.1015 |
| 42 | 483.07 | 0.44 | 0.213 | 7.82E+07 | 1.13E+09 |  | 0.1886 |  | 446.2 | 0.32 | 0.169 | 4.56E+07 | 1.88E+09 |  | 0.6066 |
| 43 | 2639.3 | 0.82 | 0.48 | 3.79E+08 | 4.66E+09 |  | 0.4956 |  | 2639.3 | 0.55 | 0.21 | 8.08E+07 | 2.96E+09 |  | 0.2212 |
| 44 | 1913.5 | 0.82 | 0.442 | 2.37E+08 | 5.13E+09 |  | 0.4189 |  | 15351 | 0.8 | 0.249 | 9.79E+08 | 1.66E+10 |  | 0.9079 |
| 45 | 695.85 | 0.54 | 0.311 | 1.30E+08 | 1.80E+09 |  | 0.2018 |  | 3737.6 | 0.35 | 0.203 | 2.46E+08 | 3.46E+09 |  | 0.8041 |
| 46 | 5439.9 | 0.81 | 0.432 | 5.42E+08 | 6.53E+09 |  | 0.3403 |  | 5439.9 | 1.03 | 0.3 | 2.32E+08 | 8.84E+09 |  | 0.365 |
| 47 | 1444.5 | 0.61 | 0.335 | 2.16E+08 | 2.27E+09 |  | 0.3406 |  | 3258.6 | 0.63 | 0.221 | 2.96E+08 | 1.24E+10 |  | 0.3103 |
| 48 | 1784.5 | 0.51 | 0.343 | 2.53E+08 | 6.92E+09 |  | 0.2191 |  | 7663.4 | 0.6 | 0.186 | 5.83E+08 | 1.64E+10 |  | 0.2809 |
| 49 | 7272.9 | 0.61 | 0.346 | 7.15E+08 | 2.55E+09 |  | 0.2131 |  | 6763.3 | 0.56 | 0.181 | 4.87E+08 | 6.95E+09 |  | 0.365 |
| 50 | 593.14 | 0.55 | 0.184 | 1.21E+08 | 1.31E+09 |  | 0.2536 |  | 856.25 | 0.7 | 0.257 | 8.44E+07 | 2.45E+09 |  | 0.4932 |
| 51 | 2287.1 | 0.85 | 0.404 | 3.63E+08 | 3.79E+09 |  | 0.3929 |  | 6357.8 | 0.85 | 0.318 | 4.21E+08 | 9.56E+09 |  | 0.4574 |
| 52 | 2175.3 | 0.76 | 0.44 | 2.80E+08 | 4.18E+09 |  | 0.2477 |  | 7540.1 | 0.64 | 0.229 | 4.99E+08 | 1.67E+10 |  | 0.4494 |
| 53 | 1161 | 0.56 | 0.313 | 1.53E+08 | 1.84E+09 |  | 0.2056 |  | 3946.6 | 0.76 | 0.259 | 3.32E+08 | 1.59E+10 |  | 0.2974 |
| 54 | 2330 | 0.82 | 0.399 | 3.59E+08 | 3.47E+09 |  | 0.2753 |  | 1076.5 | 0.36 | 0.171 | 1.53E+08 | 4.48E+09 |  | 0.4658 |
| 55 | 234.45 | 0.43 | 0.145 | 6.29E+07 | 8.16E+08 |  | 0.183 |  | 2233.8 | 0.79 | 0.239 | 1.79E+08 | 8.66E+09 |  | 0.5548 |
| 56 | 519.83 | 0.42 | 0.183 | 1.02E+08 | 1.17E+09 |  | 0.243 |  | 767.73 | 0.83 | 0.25 | 7.33E+07 | 1.06E+10 |  | 0.6223 |
| 57 | 1311.3 | 0.5 | 0.298 | 1.82E+08 | 2.44E+09 |  | 0.2712 |  | 3288.1 | 0.51 | 0.248 | 2.55E+08 | 6.51E+09 |  | 0.5279 |
| 58 | 1611.4 | 0.53 | 0.333 | 1.90E+08 | 2.73E+09 |  | 0.2744 |  | 898.8 | 0.56 | 0.299 | 1.19E+08 | 1.07E+10 |  | 0.6627 |
| 59 | 993.88 | 0.46 | 0.295 | 1.28E+08 | 1.78E+09 |  | 0.2191 |  | 8390.5 | 0.59 | 0.183 | 5.26E+08 | 9.20E+09 |  | 0.7346 |
| 60 | 1403.7 | 0.49 | 0.183 | 2.33E+08 | 1.65E+09 |  | 0.3152 |  | 3977 | 0.72 | 0.274 | 4.27E+08 | 8.58E+09 |  | 0.05859 |
| 61 | 1281.1 | 0.52 | 0.302 | 1.72E+08 | 2.39E+09 |  | 0.2992 |  | 1348.7 | 0.79 | 0.303 | 1.16E+08 | 1.53E+10 |  | 0.3516 |
| 62 | 877.12 | 0.51 | 0.29 | 1.23E+08 | 1.79E+09 |  | 0.1596 |  | 4024.5 | 0.41 | 0.118 | 3.45E+08 | 1.04E+10 |  | 0.3889 |
| 63 | 4295.8 | 0.85 | 0.476 | 4.37E+08 | 6.92E+09 |  | 0.2468 |  | 4327.4 | 0.42 | 0.131 | 3.12E+08 | 4.95E+09 |  | 0.9786 |
| 64 | 2036.9 | 0.52 | 0.311 | 2.56E+08 | 3.26E+09 |  | 0.4116 |  | 4174.2 | 0.67 | 0.256 | 3.11E+08 | 2.11E+10 |  | 0.711 |
| 65 | 382.72 | 0.46 | 0.207 | 8.07E+07 | 9.45E+08 |  | 0.2127 |  | 4550.5 | 0.61 | 0.173 | 3.13E+08 | 1.21E+10 |  | 0.6402 |
| 66 | 944.65 | 0.5 | 0.289 | 1.32E+08 | 1.92E+09 |  | 0.1828 |  | 853.96 | 0.58 | 0.226 | 7.65E+07 | 4.05E+09 |  | 0.2607 |
| 67 | 3102.6 | 0.74 | 0.394 | 3.77E+08 | 5.46E+09 |  | 0.3101 |  | 274.68 | 0.37 | 0.09 | 8.26E+07 | 8.21E+08 |  | 0.3478 |
| 68 | 473.37 | 0.39 | 0.202 | 1.02E+08 | 1.33E+09 |  | 0.2595 |  | 3319.7 | 0.52 | 0.262 | 2.78E+08 | 8.66E+09 |  | 0.3828 |
| 69 | 5362.1 | 0.56 | 0.451 | 4.62E+08 | 7.22E+09 |  | 0.2771 |  | 2151.8 | 0.87 | 0.193 | 1.85E+08 | 1.66E+10 |  | 0.3766 |
| 70 | 2547.6 | 0.62 | 0.375 | 2.57E+08 | 3.57E+09 |  | 0.2157 |  | 4232.2 | 0.59 | 0.3 | 3.93E+08 | 5.74E+09 |  | 0.3516 |
| 71 | 121.98 | 0.41 | 0.135 | 3.64E+07 | 5.29E+08 |  | 0.1155 |  | 3933.9 | 0.41 | 0.181 | 2.55E+08 | 4.21E+09 |  | 0.1374 |
| 72 | 1171.3 | 0.61 | 0.313 | 1.73E+08 | 2.04E+09 |  | 0.3255 |  | 1514.8 | 0.84 | 0.261 | 1.23E+08 | 4.73E+09 |  | 0.2224 |

# Appendix B

***Wavelet packet transform energy (WPTE)***

The wavelet packet decomposition, also called the optimal subband tree structure, is a highly effective approach for addressing non-stationary signals and transient effects. It further enhances the performance of the wavelet transform by subdividing the high-frequency component, thereby offering improved time-frequency resolution to capture the time-frequency characteristics of the signal comprehensively.

（1）The signal S can be decomposed into 2^n^ subband signals through n-level wavelet packet decomposition. such as

$S=[S_{1},S_{2},S_{3},\ldots,S_{2^{n}}]$ （B1）

The expression for the signal component at a specific level i, denoted as $S_{i}$, can be formulated as

$S_{i}=[S_{i,1},S_{i,2},S_{i,3},\ldots,S_{{i,2}^{n}}]$ （B2）

The variable i represents the frequency band (i = 1,2,... 2n), while 2^n^ signifies the total number of samples acquired within each respective band.

（2）The energy$E_{i}$ of each subband signal is computed

$E_{i}=\sum_{j=1}^{n} {||d_{i,j}||}^{2} i=1,2,\cdots,2^{n}$ （B3）

The energy of the i frequency band signal is denoted as $E_{i}$, and $||d_{i,j}||$ represents the magnitude of the j value in the i frequency band signal. $max[E_{\left( i,j \right)}]$refers to the maximum energy among all frequency bands.

（3）The total energy E of the signal can be expressed as

$E=\sum_{i=1}^{M} E_{i}$ （B4）

E represents the overall energy of the signal, while M denotes the count of signals in distinct frequency bands obtained through wavelet packet decomposition.

***Head injury criteria***

The Head Injury Criterion (HIC) is the predominant standard for assessing brain injuries and is determined through the following calculation (Prasad and Mertz 1985).

$HIC=\left[ (t_{2}-t_{1})\left( \frac{1}{t_{2}-t_{1}}\int_{t_{1}}^{t_{2}} \alpha(t)dt \right)^{2.5} \right]_{\max}$ （B5）

The variable $\alpha(t)$ represents the resultant linear acceleration of the head center of mass during a collision while denoting the $t$ interval at which the HIC reaches its maximum value. In pedestrian collisions, this time interval is 15ms.

The Brain Injury Criteria (BrIC) was developed in 2013 by Takhounts et al (Takhounts et al., 2013). who studied head diffuse brain injury among American football players following collisions. They proposed the BrIC injury criteria, which can be expressed through the following formula.

$BrIC=\sqrt{(\frac{\omega_{x}}{\omega_{\mathrm{xC}}})^{2}+(\frac{\omega_{y}}{\omega_{\mathrm{yC}}})^{2}+(\frac{\omega_{z}}{\omega_{\mathrm{zC}}})^{2}}$ （B6）

Where $\omega_{x}$, $\omega_{y}$, and $\omega_{z}$ denote the time-varying angular velocity peaks in each anatomical direction, while $\omega_{\mathrm{xC}}$, $\omega_{\mathrm{yC}}$, and$\omega_{\mathrm{zC}}$ represent the corresponding critical values of 66.2rad/s, 59.1rad/s, and 44.25rad/s respectively as determined by experimental data from pendulum dummy collisions.

The Diffuse Axonal Multi-Axis General Evaluation (DAMAGE) was proposed in 2019 by Gabler et al (Gabler et al., 2019). to quantify the maximum brain strain across various impact conditions. It is based on a second-order mechanical system and utilizes three physical mass-spring-damping element simulations. The parameters for effective mass, stiffness, and damping were determined by subjecting a finite element model of an adult male at the 50th percentile to rotational pulse multiaxis testing. Prediction of maximal mental fatigue was based on the angular acceleration time history associated with head impacts.

$DAMAGE=\beta{max}_{t}\{\mid\delta(t)\mid$ （B7）

The vector $\delta(t)$ represents the temporal deformation history of the brain during rotational motion around each axis of the head, while β is a scaling factor that establishes the relationship between the maximum synthetic displacement of the system and MPS values derived from the FE brain model.

# Appendix C

**Note:** rmTBI: repetitive mild traumatic brain injury, rsTBI: repetitive severe traumatic brain injury.


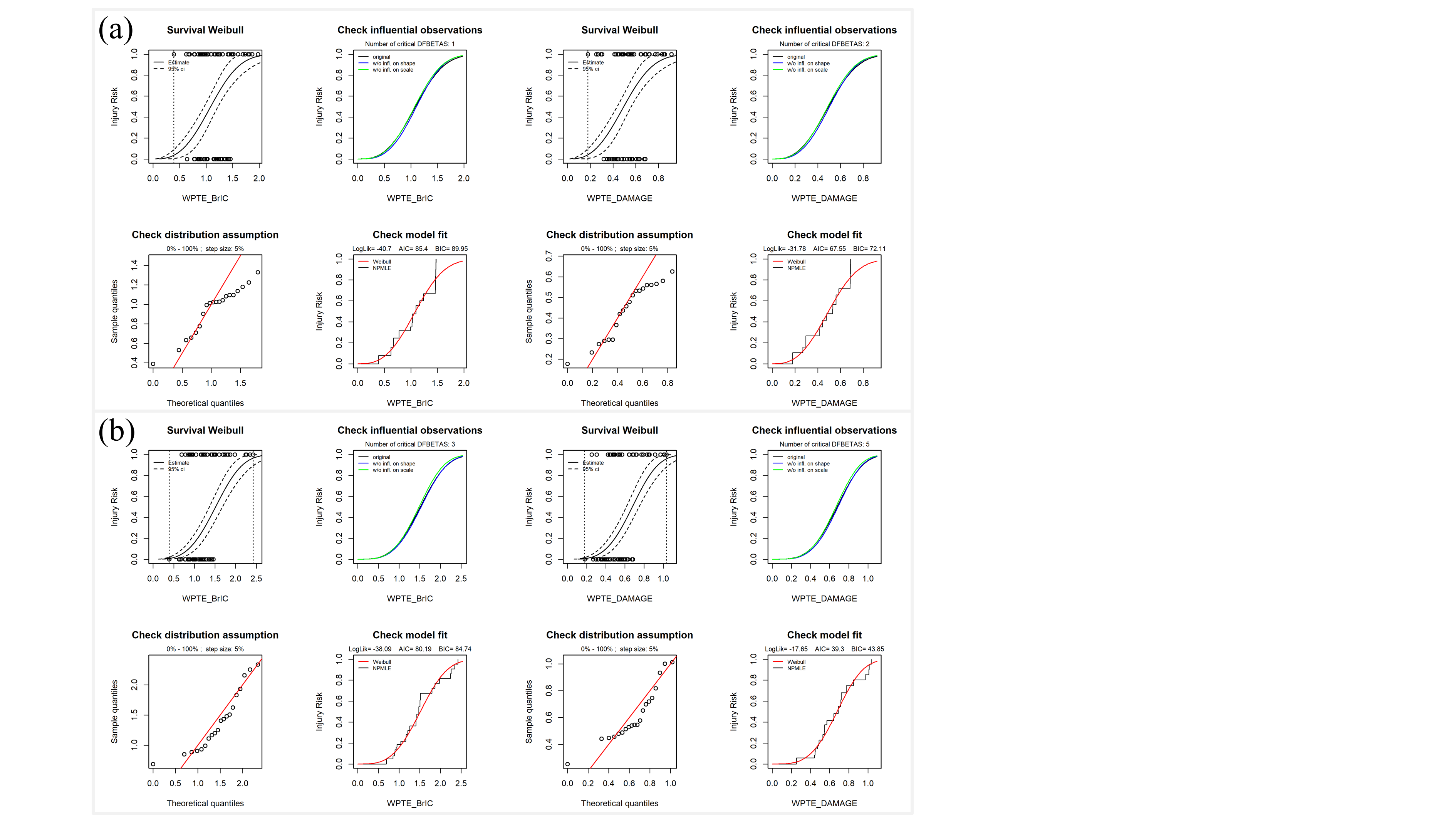


**Fig. C1.** IRFs based on WPTE_BrIC and WPTE_DAMAGE. (a) rmTBI; (b) rsTBI.


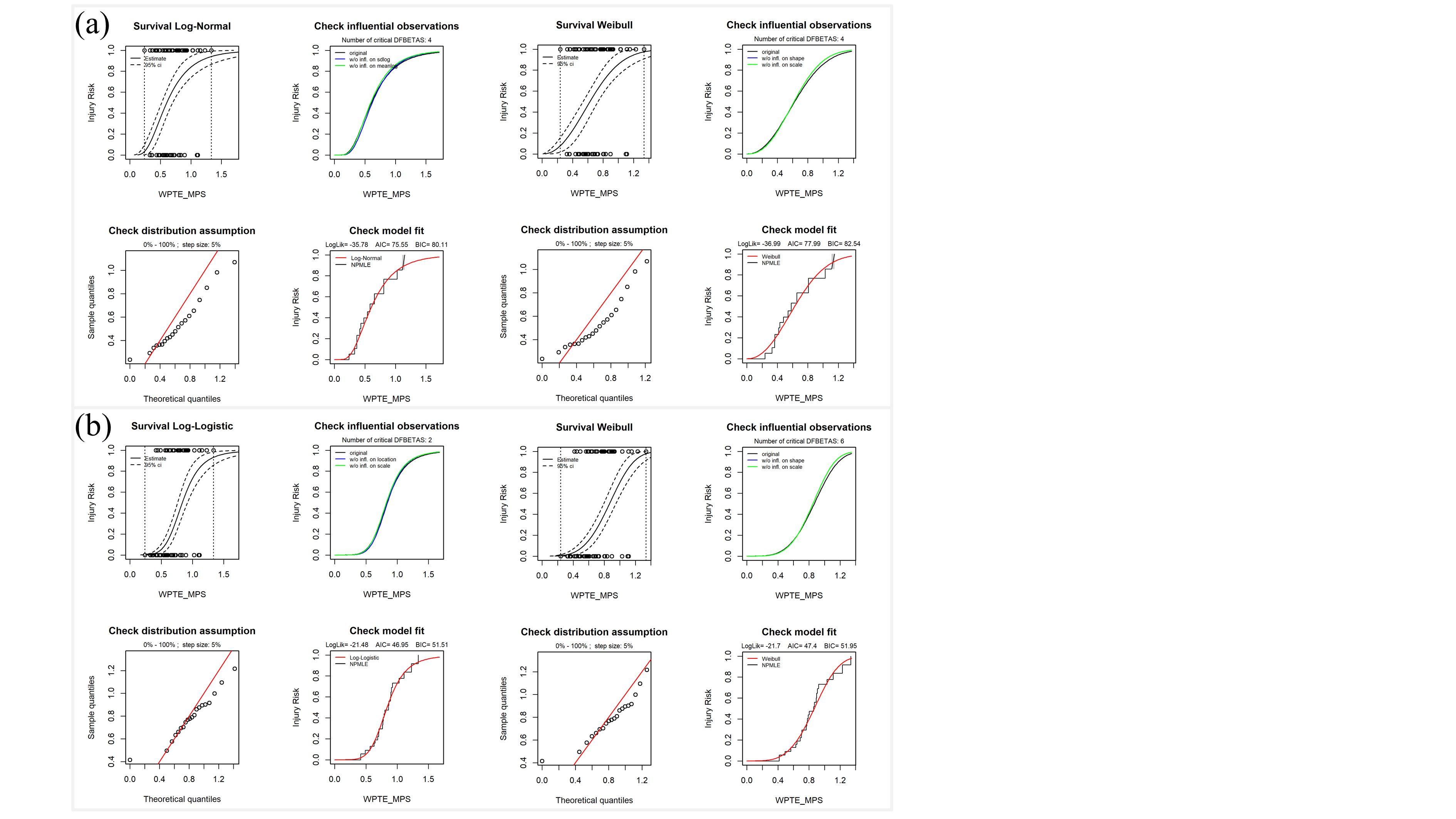


**Fig. C2**. IRFs of WPTE_MPS based on optimal distribution and Weibull distribution. (a) rmTBI; (b) rsTBI

**Table C1.** Summary of the measures and NCIS results of the Repetitive Mild Traumatic Brain Injury Risk Assessment Function

| Metrics used in the repetitive brain injury risk assessment function |  | 95% Confidence intervals | |  |  |  |
| --- | --- | --- | --- | --- | --- | --- |
|  | Risk Level | Lower bound | Upper bound | Mean value | NCIS | Quality index |
| WPTE_BrIC | 5% | 0.29 | 0.66 | 0.44 | 0.84 | fair |
|  | 10% | 0.41 | 0.77 | 0.57 | 0.63 | fair |
|  | 25% | 0.66 | 0.97 | 0.80 | 0.39 | good |
|  | 50% | 0.96 | 1.22 | 1.08 | 0.24 | good |
|  | 75% | 1.22 | 1.56 | 1.38 | 0.25 | good |
|  | 90% | 1.39 | 1.94 | 1.64 | 0.34 | good |
|  | 95% | 1.49 | 2.18 | 1.80 | 0.38 | good |
| WPTE_DAMAGE | 5% | 0.13 | 0.29 | 0.19 | 0.84 | fair |
|  | 10% | 0.18 | 0.35 | 0.25 | 0.68 | fair |
|  | 25% | 0.29 | 0.44 | 0.36 | 0.42 | good |
|  | 50% | 0.44 | 0.56 | 0.49 | 0.24 | good |
|  | 75% | 0.56 | 0.72 | 0.63 | 0.25 | good |
|  | 90% | 0.64 | 0.90 | 0.76 | 0.34 | good |
|  | 95% | 0.69 | 1.02 | 0.84 | 0.39 | good |
| WPTE_MPS | 5% | 0.19 | 0.36 | 0.26 | 0.65 | fair |
|  | 10% | 0.24 | 0.41 | 0.31 | 0.55 | fair |
|  | 25% | 0.35 | 0.52 | 0.43 | 0.40 | good |
|  | 50% | 0.52 | 0.70 | 0.60 | 0.30 | good |
|  | 75% | 0.71 | 1.02 | 0.85 | 0.36 | good |
|  | 90% | 0.90 | 1.49 | 1.16 | 0.51 | fair |
|  | 95% | 1.03 | 1.89 | 1.39 | 0.62 | fair |

**Table C2.** Summary of the measures and NCIS results of the Repetitive Severe Traumatic Brain Injury Risk Assessment Function

| Metrics used in the repetitive brain injury risk assessment function |  | 95% Confidence intervals | |  |  |  |
| --- | --- | --- | --- | --- | --- | --- |
|  | Risk Level | Lower bound | Upper bound | Mean value | NCIS | Quality index |
| WPTE_BrIC | 5% | 0.55 | 0.88 | 0.70 | 0.47 | good |
|  | 10% | 0.71 | 1.04 | 0.86 | 0.38 | good |
|  | 25% | 1.02 | 1.33 | 1.16 | 0.27 | good |
|  | 50% | 1.36 | 1.68 | 1.51 | 0.21 | good |
|  | 75% | 1.67 | 2.07 | 1.86 | 0.22 | good |
|  | 90% | 1.91 | 2.45 | 2.17 | 0.25 | good |
|  | 95% | 2.05 | 2.68 | 2.34 | 0.27 | good |
| WPTE_DAMAGE | 5% | 0.26 | 0.41 | 0.33 | 0.45 | good |
|  | 10% | 0.34 | 0.48 | 0.40 | 0.35 | good |
|  | 25% | 0.47 | 0.60 | 0.53 | 0.25 | good |
|  | 50% | 0.61 | 0.75 | 0.68 | 0.21 | good |
|  | 75% | 0.74 | 0.91 | 0.82 | 0.21 | good |
|  | 90% | 0.84 | 1.06 | 0.95 | 0.23 | good |
|  | 95% | 0.90 | 1.15 | 1.02 | 0.25 | good |
| WPTE_MPS | 5% | 0.42 | 0.59 | 0.50 | 0.34 | good |
|  | 10% | 0.49 | 0.65 | 0.57 | 0.28 | good |
|  | 25% | 0.62 | 0.76 | 0.69 | 0.20 | good |
|  | 50% | 0.76 | 0.92 | 0.84 | 0.19 | good |
|  | 75% | 0.90 | 1.15 | 1.02 | 0.25 | good |
|  | 90% | 1.05 | 1.46 | 1.24 | 0.33 | good |
|  | 95% | 1.16 | 1.73 | 1.42 | 0.40 | good |

QI: based on the study by Petitjean et al. (2009), we can categorize the quality index of IRFs into four types: 'good'(0-0.5); 'fair'(0.5-1.0); 'margina'(1.0-1.5); 'Unacceptable'(>1.5).

**Table C3**. Parameter output for survival analysis with age covariates

| Injury | Metrics | β0(Intercept) | β1(Age) | Scale |
| --- | --- | --- | --- | --- |
| rmTBI | WPTE_BrIC | 0.621 | -0.0069 | 0.352 |
|  | WPTE_DAMAGE | -0.186 | -0.0065 | 0.375 |
|  | WPTE_MPS | 0.113 | -0.0068 | 0.478 |
| rsTBI | WPTE_BrIC | 0.854 | -0.0052 | 0.303 |
|  | WPTE_DAMAGE | -0.041 | -0.0038 | 0.282 |
|  | WPTE_MPS | 0.207 | -0.004 | 0.259 |

**Table C4.** Model coefficient and quality index based on Weibull distribution IRFs

| Metrics | Injury | Scale(α) | Shape(β) | 5%Risk(QI) | 25%Risk(QI) | 50%Risk(QI) | AIC |
| --- | --- | --- | --- | --- | --- | --- | --- |
| WPTE_MPS | rmTBI | 0.741 | 2.207 | 0.193(0.87) | 0.421(0.51) | 0.628(0.31) | 77.99 |
|  | rsTBI | 0.952 | 3.915 | 0.446(0.43) | 0.692(0.24) | 0.867(0.18) | 47.4 |

**References**

Gabler, L. F., Crandall, J. R., and Panzer, M. B. (2019). Development of a second-order system for rapid estimation of maximum brain strain. Ann Biomed Eng. 47(9):1971-1981.

Petitjean A, Trosseille X, Petit P, Irwin A, Hassan J, Praxl N. 2009. Injury risk curves for the WorldSID 50th male dummy (No. 2009-22-0016). SAE Tech. Pap.

Prasad, P., and Mertz, H. J. (1985). The position of the United States delegation to the ISO Working Group 6 on the use of HIC in the automotive environment. SAE transactions, 106-116.

Takhounts, E. G., Craig, M. J., Moorhouse, K., McFadden, J., and Hasija, V. (2013). Development of brain injury criteria (BrIC) (No. 2013-22-0010). SAE Technical Paper.
